# Identification of a Novel Substrate for eEF2K and the AURKA‐SOX8 as the Related Pathway in TNBC

**DOI:** 10.1002/advs.202412985

**Published:** 2025-02-14

**Authors:** Xiaoya Wan, Rong Gong, Xiaobao Zhao, Yizhi Li, Tianjiao Shan, Changxin Zhong, Rongfeng Zhu, Zonglin Chen, Shilong Jiang, Linhao He, Shijun Cao, Sheng Tian, Jinming Yang, Na Ye, Wenjun Yi, Yan Cheng

**Affiliations:** ^1^ Department of Pharmacy The Second Xiangya Hospital Central South University Changsha 410011 China; ^2^ Hunan Provincial Engineering Research Centre of Translational Medicine and Innovative Drug Changsha 410011 China; ^3^ Department of Medicinal Chemistry Jiangsu Key Laboratory of Neuropsychiatric Diseases and College of Pharmaceutical Sciences Soochow University Suzhou 215123 China; ^4^ Department of General Surgery The Second Xiangya Hospital Central South University Changsha 410011 China; ^5^ Department of Pharmacy Xiangya Hospital Central South University Changsha 410028 China; ^6^ Department of Cancer Biology and Toxicology Department of Pharmacology College of Medicine and Markey Cancer Center University of Kentucky Lexington KY 40536 USA; ^7^ Jiangsu Province Engineering Research Center of Precision Diagnostics and Therapeutics Development Soochow University Suzhou 215123 China; ^8^ Clinical Research Center For Breast Disease In Hunan Province Changsha 410011 China; ^9^ FuRong Laboratory Changsha Hunan 410078 China; ^10^ Key Laboratory of Diabetes Immunology Central South University Ministry of Education Changsha 410011 China; ^11^ NHC Key Laboratory of Cancer Proteomics & State Local Joint Engineering Laboratory for Anticancer Drugs Xiangya Hospital Central South University Changsha 410008 China

**Keywords:** AURKA, eEF2K, eEF2K degrader, SOX8, triple‐negative breast cancer

## Abstract

Eukaryotic elongation factor 2 kinase (eEF2K) has been considered as a putative target for cancer therapy; however, the underlying mechanisms of eEF2K in triple‐negative breast cancer (TNBC) progression remain to be fully elucidated. In this study, it is shown that eEF2K is highly expressed in TNBC and is associated with poor prognosis. In vitro, in vivo, and patient‐derived organoid experiments demonstrate that knockdown of eEF2K significantly impedes progression of TNBC. Proteomic analysis and confirmation experiments reveal that eEF2K positively regulates the mRNA and protein expressions of sex‐determining region Y‐box 8 (SOX8). Mechanistically, eEF2K binds to and phosphorylates aurora kinase A (AURKA) at S391, a newly identified phosphorylation site critical for maintaining AURKA protein stability and kinase activity. Moreover, the compound C1, a molecular glue to degrade eEF2K, is optimized by designing and synthesizing its derivatives using reasonable structure‐based optimization approach. The new compound C4 shows  better ability to degrade eEF2K and stronger anti‐cancer activity than C1. These findings not only uncover the pivotal role of the eEF2K/AURKA/SOX8 axis in TNBC progression, but also provide a promising lead compound for developing novel drug for treatment of TNBC.

## Graphical abstract

This study reports that eukaryotic elongation factor 2 kinase (eEF2K) is a key regulator in promoting the progression of triple‐negative breast cancer (TNBC). Mechanistically, the authors identified AURKA as a novel substrate for eEF2K. In addition, the authors developed a new small molecule compound C4, which acts as a molecular glue for eEF2K and can significantly alleviate the malignant phenotype of TNBC.

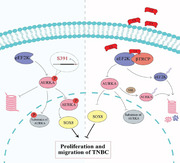

## 1 Introduction

Breast cancer is the type of tumor with the highest incidence in women,^[^ ^1^ ^]^ and recurrence and metastasis are the leading causes of death in breast cancer patients.^[^ ^2^ ^]^ Triple‐negative breast cancer (TNBC) is defined by lacking the clinicopathological expressions of estrogen receptor (ER), progesterone receptor (PR) and human epidermal growth factor receptor 2 (HER2), and accounts for ≈15% of breast cancer incidence.^[^ ^3^, ^4^ ^]^ Due to its high degree of malignancy and aggressiveness, TNBC is responsible for ≈30% of breast cancer‐related deaths.^[^ ^5^, ^6^ ^]^ Lack of therapeutic targets is the main reason why TNBC is so challenging to treat.^[^ ^7^ ^]^ Therefore, there is an urgent need to identify effective targets to inhibit TNBC growth and metastasis.

Eukaryotic elongation factor 2 Kinase (eEF2K), an atypical protein kinase involved in calmodulin‐mediated signaling pathways, has been found to be highly expressed in various solid tumors, including breast cancer, and its high expression is associated with poor survival of patients.^[^ ^8^ ^]^ It has been reported that miR‐34a, miR‐603, or miR‐22‐3p directly binds to the 3′‐untranslated region of eEF2K mRNA and suppresses its expression, leading to inhibition of TNBC cell proliferation, motility and invasion.^[^ ^9^, ^10^, ^11^ ^]^ Transcription factor FOXM1 promotes the progression of TNBC by up‐regulating eEF2K gene expression.^[^ ^12^ ^]^ Additionally, genetic or pharmacological inhibition of eEF2K synergistically inhibits the growth of TNBC cells with glutaminase inhibitors or 4EBP1 deletion.^[^ ^13^ ^]^ In addition to the classical substrate eEF2, recent studies have revealed new substrates of eEF2K in tumors. Deng et al. reported that eEF2K phosphorylates the Tyr705 site of STAT3, promoting the transcription of SPP1 and the progression of melanoma.^[^ ^14^ ^]^ Our previous study demonstrated that eEF2K stabilizes PD‐L1 by directly phosphorylating GSK3β at the Ser9 site, resulting in tumor immune escape.^[^ ^15^ ^]^ In addition, we identified the compound C1 as the first molecular glue of eEF2K.^[^ ^16^ ^]^

SOX8, a member of the transcription factor sex‐determining region Y‐box (SOX) family, binds to the promoter regions of some cancer‐promoting factors such as EZH2, FOXK1, FZD6, FZD7, and GOLPH3, promoting chemotherapy resistance, tumor progression, and epithelial‐to‐mesenchymal transition (EMT).^[^ ^17^, ^18^, ^19^, ^20^ ^]^ SOX8 is abnormally expressed in a variety of tumors including breast cancer, and has been shown to be one of the most upregulated genes and a crucial regulator in TNBC.^[^ ^21^, ^22^, ^23^ ^]^ Aurora kinase A (AURKA), a key upstream regulator of SOX8, can directly phosphorylate Ser327 site of SOX8 to enhance its activity.^[^ ^19^ ^]^ In addition, AURKA up‐regulates c‐Myc by enhancing its protein stability, which in turn promotes SOX8 transcription by binding to its promoter.^[^ ^19^, ^24^ ^]^ In this study, we show that eEF2K is not only highly expressed and is associated with poor prognosis in TNBC, but  also directly phosphorylates S391 site of AURKA, enhancing AURKA protein stability and kinase activity, thereby up‐regulating the expression of SOX8. In addition, compound C4, obtained through structural modification and optimization based on our newly developed eEF2K degrader compound C1, shows a significant inhibitory effect on TNBC via targeting eEF2K. This study suggests that targeting eEF2K may present a promising strategy for treating TNBC.

## 2 Results

### 2.1 eEF2K is Highly Expressed in TNBC and is Associated with Cancer Progression and Poor Prognosis

**Figure 1:**
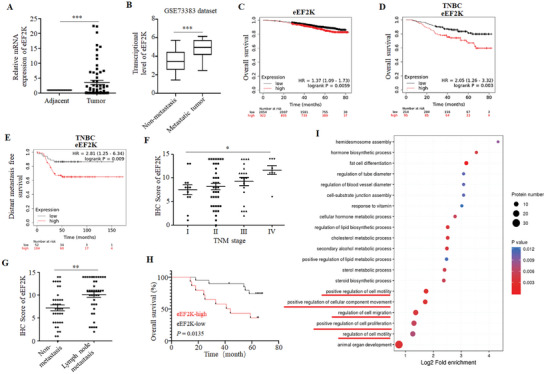
eEF2K is highly expressed in TNBC and is associated with cancer progression and poor prognosis. A) QRT‐PCR analyses of eEF2K mRNA expression in 64 paired breast cancer and adjacent non‐tumor tissues. GAPDH was the internal control. ***P < 0.001. B) The expressions of eEF2K in patients from GSE73383 breast cancer cohorts. ***P < 0.001. C) The correlation between eEF2K expression and overall survival (OS) in breast cancer patients was analyzed by online Kaplan‐Meier plotter website. D,E) The correlations between eEF2K expression and OS (D) or distant metastasis free survival (DMFS) (E) in TNBC patients were analyzed by online Kaplan‐Meier plotter website. F) The relationship between eEF2K expression and TNM stage, including 12 cases of stage I, 39 cases of stage II, 21 cases of stage III, and 8 cases of stage IV. *P < 0.05. G) The expression of eEF2K in breast cancer with (n = 41) or without (n = 39) lymph node metastasis was analyzed based on IHC scores. **P < 0.01. H) The OS in TNBC patients with low or high eEF2K expression was analyzed by Kaplan–Meier curve. I) Proteomic analysis of eEF2K‐silenced MDA‐MB‐231 cells, followed by gene ontology enrichment analysis.

To determine the importance of eEF2K in breast cancer, we examined the mRNA expressions of eEF2K in 64 pairs of breast cancer and adjacent non‐malignant tissues using quantitative real time polymerase chain reaction (qRT‐PCR). **Figure** **1**A showed that eEF2K expression was remarkably higher in breast cancer tissues than that in the paired adjacent normal tissues. GEO dataset (GSE73383) analysis revealed that mRNA levels of eEF2K in the metastatic breast cancers were significantly higher than those in the non‐metastatic breast cancers (Figure 1B). The online Kaplan‐Meier Plotter database (http://kmplot.com/analysis/) showed that eEF2K expression was associated with poor survival in breast cancer patients (Figure 1C). Further, there was a significant relationship between higher expression of eEF2K and worse overall survival (OS) (Figure 1D; Figure S1A, Supporting Information) and distant metastasis‐free survival (DMFS) (Figure 1E; Figure S1B, Supporting Information) in TNBC as compared to other subtypes of breast cancer. These results indicate that breast cancer patients with high eEF2K expression are more likely to suffer from distant metastasis and have a shorter survival, especially for those with TNBC.

To further explore the role and clinical implication of eEF2K in TNBC, we performed immunohistochemistry (IHC) on 80 TNBC specimens, and the associations between eEF2K levels and the indicated clinicopathological characteristics are shown in Table S3 (Supporting Information); eEF2K protein expression was positively correlated with lymph node metastasis (*P* < 0.0001), distant metastasis (*P* = 0.0136), tumor‐node‐metastasis (TNM) stage (*P* = 0.0021) and tumor size (maximum diameter) (*P* = 0.0021), indicating that eEF2K may play an important role in modulating proliferation and metastasis of TNBC. Furthermore, expressions of eEF2K in stage IV TNBC were higher than those in stage I TNBC (Figure 1F). The expressions of eEF2K in patients with lymph node metastasis were significantly higher than those with negative lymph node (Figure 1G). The Kaplan‐Meier analysis revealed that patients with high eEF2K expression exhibited unfavorable OS (Figure 1H). Given the clinical implication of eEF2K in TNBC, we performed proteomic sequencing of eEF2K‐silenced TNBC cell line MDA‐MB‐231, and our GO enrichment analysis showed that eEF2K was involved in signaling pathways related to cell proliferation and migration (Figure 1I). We further examined endogenous eEF2K expression in five TNBC cell lines and one normal mammary epithelial cell line MCF‐10A, and found that eEF2K was slightly expressed in MCF‐10A, but was highly expressed in TNBC cell lines, especially in MDA‐MB‐231 and HCC1806 cells (Figure S1C, Supporting Information). These data support the oncogenic role of eEF2K in TNBC progression

### 2.2 Silencing of eEF2K Significantly Inhibits the Malignant Phenotype of TNBC

**Figure 2:**
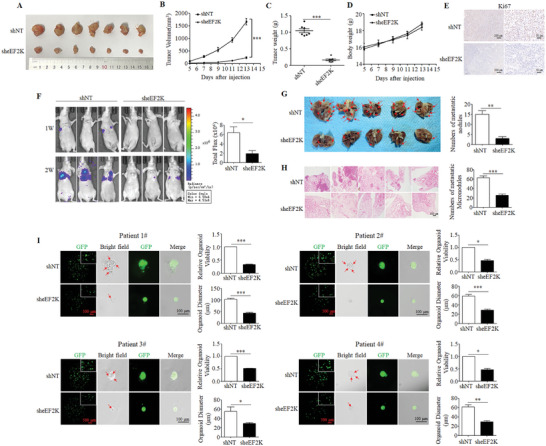
Silencing of eEF2K significantly inhibits the malignant phenotype of TNBC. A) Subcutaneous tumors were excised and photographs were taken at the termination of the experiment. B) Volume of tumors was measured on the days as indicated. ***P < 0.001. C) The tumor weight was measured after the experiment. Data were represented as mean ± SD. ***P < 0.001. D) The body weight was measured after the experiment. E) IHC was performed to determine the levels of Ki67 in xenograft tumors. Scale bar (left), 200 µm. Scale bar (right), 50 µm. F) Representative bioluminescence images of metastasis tumors. *P < 0.05. G) Representative photographs of lung metastases were obtained from nude mice, **P < 0.01. H) H&E staining of lung metastatic tumors. Scale bar, 500 µm. ***P < 0.001. I) Adenosine triphosphate bioluminescence assay was used to detect the viability of four PDOs after eEF2K silencing. Red scale bar, 500 µm; Black scale bar, 100 µm. ***P < 0.001, **P < 0.01, *P < 0.05.

To verify whether eEF2K is a critical regulator of the progression of TNBC, MDA‐MB‐231, and HCC1806 cells were transfected with eEF2K shRNA to establish stable eEF2K knockdown cells (Figure S2A, Supporting Information). We found that knockdown of eEF2K significantly inhibited cell proliferation, as indicated by the decreased cell numbers, colony numbers, and 5‐Ethynyl‐2′‐deoxyuridine (EdU)‐positive cells in eEF2K shRNA cells as compared with control cells (Figure S2B–D, Supporting Information). Wound‐healing assay showed that eEF2K depletion substantially inhibited the wound closure rate in both MDA‐MB‐231 and HCC1806 cells (inhibitory rate is ≈70%) (Figure S2E, Supporting Information). The inhibition rate of eEF2K knockdown on cell proliferation was ≈30% under the same conditions, which was much lower than its inhibitory effect on cell migration (Figure S2F, Supporting Information). The transwell assay showed that the numbers of migrating and invasive cells were markedly reduced upon eEF2K silencing (Figure S2G, Supporting Information). As shown in Figure S2H (Supporting Information), knockdown of eEF2K significantly decreased the expressions of interstitial markers, N‐cadherin, and vimentin, but increased the expression of epithelial marker E‐cadherin (Figure S2H,I, Supporting Information). These results suggest that eEF2K may accelerate TNBC metastasis by promoting EMT. Animal experiments showed that the tumor volume and weight were significantly reduced in the sheEF2K group as compared to the shNT group (**Figure** **2**A–C), but the body weight of the mice had no significant changes (Figure 2D). The positive rate of Ki67 in the tumor was markedly decreased in the sheEF2K group (Figure 2E).

Next, we injected luciferase‐expressing shNT‐ or sheEF2K‐MDA‐MB‐231 cells into the tail vein of BALB/C mice to determine the effect of eEF2K on breast cancer metastasis. We observed that silencing of eEF2K significantly decreased TNBC metastasis (Figure 2F) and the number of metastatic nodules in the lungs (Figure 2G). Hematoxylin‐eosin (H&E) staining of lung tissues also showed a remarkable reduction of lung metastases in the sheEF2K group (Figure 2H). These results indicate that depletion of eEF2K inhibits tumor growth and metastasis both in vitro and in vivo.

Further, we established the patient‐derived organoids (PDOs) using tumor tissues obtained from TNBC patients (Figure S2J, Supporting Information), and then infected them with GFP‐eEF2K shRNA and GFP control lentivirus, respectively (Figure S2K, Supporting Information). There were a smaller diameter of PDOs and a lower proportion of budding structures (red arrow) in the sheEF2K group, as compared to the control group (Figure 2I). Adenosine triphosphate bioluminescence assay showed that organoid activity was significantly decreased after eEF2K silencing (Figure 2I). These results confirm that depletion of eEF2K can significantly modulate the malignant phenotype of TNBC.

### 2.3 eEF2K Promotes the Malignant Phenotype of TNBC through Increasing the Expression of SOX8

**Figure 3:**
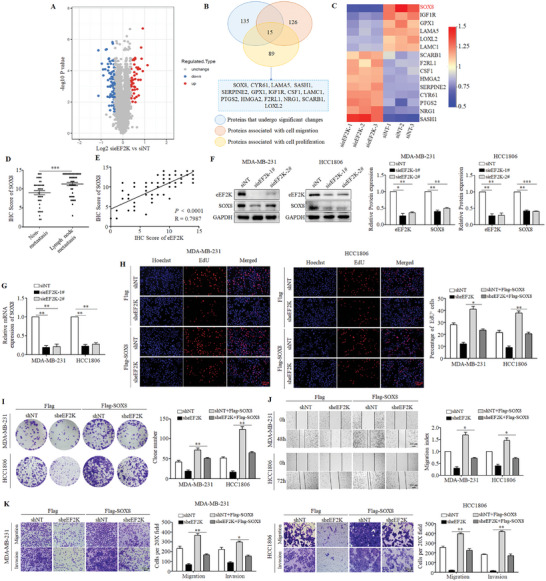
eEF2K promotes the malignant phenotype of TNBC through up‐regulating SOX8. A) Proteomic analysis of eEF2K‐silenced MDA‐MB‐231 cells, followed by the presentation of a volcano plot to illustrate proteins with significant alterations. B) The Venn diagram shows intersecting targets obtained by 135 significantly altered proteins, 126 proteins associated with cell migration, and 89 proteins associated with cell proliferation. C) Heatmap of 15 intersecting proteins. D) The expression of SOX8 in breast cancer with (n = 41) or without (n = 39) lymph node metastasis was analyzed based on immunohistochemical scores. ***P < 0.001. E) The correlation between eEF2K and SOX8 was analyzed based on immunohistochemical scores of tissue microarray. F) MDA‐MB‐231 and HCC1806 cells were transfected with a non‐targeting siRNA or eEF2K siRNAs. The protein expressions of eEF2K and SOX8 were measured by western blot. ***P < 0.001, **P < 0.01, *P < 0.05. G) The mRNA expressions of SOX8 were measured by qRT‐PCR. **P < 0.01. MDA‐MB‐231 and HCC1806 cells stably expressing the eEF2K‐targeted shRNA and the non‐target control were transfected with Flag‐SOX8 expression plasmid; H) EdU staining assays were conducted to assess the proliferation ability. Scale bar, 100 µm. **P < 0.01, *P < 0.05. I) Colony formation assays were applied to evaluate cell proliferation ability. **P < 0.01. J) Wound healing assays were used to assess cell migration ability. Scale bar, 200 µm. *P < 0.05. K) Transwell assays were performed to evaluate the migration and invasion abilities. Scale bar, 100 µm. **P < 0.01, *P < 0.05.

To determine the molecular mechanism by which eEF2K promotes TNBC progression, our proteomic analysis showed that eEF2K silencing resulted in significant up‐regulation of 45 proteins and down‐regulation of 90 proteins (proteins were clustered at the 1.3‐fold changes with a *P* value less than 0.05) (**Figure** **3**A). By intersecting these 135 significantly altered proteins, 126 proteins are related to cell migration, and 89 proteins are related to cell proliferation, and Venn diagram revealed 15 intersecting protein targets (Figure 3B). As shown in Figure 3C, among those proteins, SOX8 showed the most dramatic down‐regulation following eEF2K silencing. Analysis of the qRT‐PCR results of 64 paired human breast cancer and adjacent non‐tumor tissues found that mRNA expressions of SOX8 were significantly up‐regulated in breast cancer tissues, as compared to the corresponding non‐tumor tissues (Figure S3A, Supporting Information). There was a significant positive correlation between the mRNA expressions of eEF2K and SOX8 with a Pearson correlation coefficient (R) of 0.4481 (Figure S3B, Supporting Information). Prognostic analysis based on the Kaplan‐Meier Plotter database showed that the high expression of SOX8 was associated with poor prognosis of breast cancer (Figure S3C, Supporting Information). In‐depth analysis revealed that there was a more significant correlation between high expression of SOX8 and worse OS and DMFS in TNBC compared to other subtypes of breast cancer (Figure S3D,E, Supporting Information).

We next analyzed the expression of SOX8 in different breast cancer subtypes using GEO dataset and LinkedOmics website. As shown in Figure S3F,G (Supporting Information), TNBC has the highest expression of SOX8 compared to other subtypes of breast cancer. Analysis of tissue microarray of TNBC patient samples found that the expression of SOX8 was positively associated with the lymph node metastasis (*P* = 0.0026), distant metastasis (*P* = 0.0046), and higher TNM stage (*P* = 0.0367) (Table S4, Supporting Information). As shown in Figure 3D, the expression of SOX8 was significantly higher in TNBC patients with the lymph node metastasis (41 of 80) than that in those with negative lymph nodes (39 of 80). In addition, the expressions of SOX8 in patients with stage IV TNBC were higher than that in those with stage I TNBC (Figure S3H, Supporting Information). Further, there was a significant positive correlation between the expressions of eEF2K and SOX8 in TNBC tissue microarray (IHC score: R = 0.7987) (Figure 3E).

We also showed that the protein and mRNA levels of SOX8 were significantly decreased when eEF2K was silenced in TNBC cells (Figure 3F,G). In contrast, overexpression of eEF2K up‐regulated the protein and mRNA levels of SOX8 in TNBC cells (Figure S3I,J, Supporting Information). The reduced protein levels of N‐cadherin and vimentin, as well as the up‐regulation of E‐cadherin in the cells with eEF2K knockdown were reversed by SOX8 overexpression (Figure S3K, Supporting Information). SOX8 overexpression also rescued the inhibition of cell proliferation, migration, and invasion caused by eEF2K knockdown in TNBC cells (Figure 3H–K). These results indicate that SOX8 is a downstream player of eEF2K, and mediates the effect of eEF2K on the malignant phenotype of TNBC.

### 2.4 eEF2K Directly Binds with and Maintains Protein Stability of AURKA

**Figure 4:**
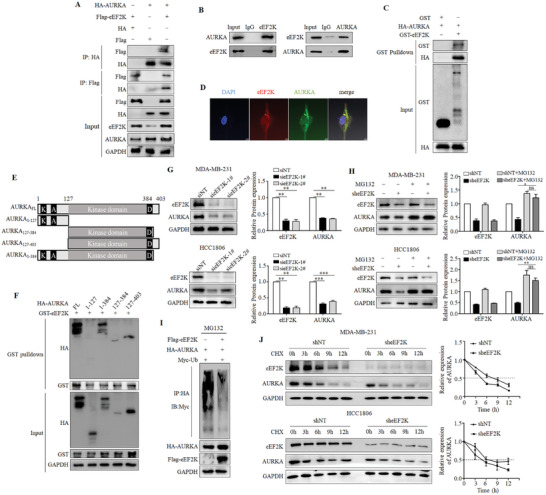
eEF2K directly binds with AURKA and maintains its stability. A) HEK293T cells were transfected with Flag or Flag‐eEF2K and HA or HA‐AURKA plasmids, and then subjected to immunoprecipitation with anti‐Flag or anti‐HA antibodies. The lysates and immunoprecipitates were then blotted. B) Endogenous eEF2K/AURKA proteins were immunoprecipitated with the anti‐AURKA or anti‐eEF2K antibody, respectively. The immunocomplex was detected by western blot in MDA‐MB‐231 cells. C) HEK293T cells were transfected with HA‐AURKA and then subjected to immunoprecipitation with anti‐HA antibodies. Purified recombinant GST and GST‐eEF2K proteins interacted with immune complexes. GST‐eEF2K and GST proteins were pulled down with glutathione beads. HA was detected by western blot. D) The cellular location of eEF2K and AURKA was examined in MDA‐MB‐231 cells by immunofluorescence staining. Scale bar, 10 µm. E) Schematic diagram of AURKA constructs used in this study. F) Immunoprecipitation experiments were performed with purified GST‐eEF2K and HEK293T cell lysates exogenously expressing the full‐length or different functional domains of HA‐AURKA. The HA was detected by western blot analysis. G) The expression of AURKA protein after silencing eEF2K was detected by western blot assay. ***P < 0.001, **P < 0.01. H) MDA‐MB‐231 and HCC1806 cells stably expressing the eEF2K‐targeted shRNA or the non‐target control were treated with 20 µm MG132 for 6 h. The expressions of eEF2K and AURKA were measured by western blot. ns, no significance. **P < 0.01, *P < 0.05. I) HEK293T cells transfected with the indicated constructs were treated with MG132 (20 µm) for 6 h before harvest. Ubiquitination of HA‐AURKA was immunoprecipitated with anti‐Myc antibodies. J) MDA‐MB‐231 and HCC1806 cells stably expressing the eEF2K‐targeted shRNA and the non‐target control were treated with CHX (10 µg ml^−1^) for the indicated time. AURKA was detected by western blot.

Next, we sought to explore the underlying mechanisms by which eEF2K regulates SOX8 expression. Immunoprecipitation assay demonstrated that eEF2K was not physically associated with SOX8 (Figure S4A, Supporting Information). To analyze how eEF2K regulates SOX8, we carried out Immunoprecipitation‐Mass Spectrometry assay and found that AURKA, an oncogenic protein that promotes SOX8 expression, interacted with eEF2K (Figure S4B,C, Supporting Information). The known eEF2K‐interating proteins such as eEF2 and Homer1, were also precipitated with anti‐Flag antibodies. To verify this result, we co‐transfected HEK293T cells with Flag or Flag‐eEF2K, and HA or HA‐AURKA plasmids, and then performed co‐immunoprecipitation experiments with anti‐Flag antibodies or anti‐HA antibodies. Western blot showed the physical binding between eEF2K and AURKA in these cells (**Figure** **4**A). Figure 4B demonstrates the endogenous interaction of AURKA with eEF2K in MDA‐MB‐231 cells. Furthermore, GST‐pulldown experiment showed that the purified GST‐eEF2K protein but not GST, formed a complex with AURKA, indicating an interaction between AURKA and eEF2K (Figure 4C). Immunofluorescence experiments exhibited the co‐localization of AURKA and eEF2K in MDA‐MB‐231 cells (Figure 4D). We next identified the terminal regulatory domain (aa 384–403) and central catalytic domain (aa 127–384) of AURKA that interact with eEF2K (Figure 4E,F). Overall, these results demonstrate a direct binding of eEF2K with AURKA. We also demonstrated that silencing of eEF2K markedly decreased the protein expression of AURKA in TNBC cells (Figure 4G), but had no effect on its mRNA level (Figure S4D, Supporting Information). MG123, a proteasome inhibitor, rescued the decreased expression of AURKA in the eEF2K‐silenced cells (Figure 4H). The ubiquitination experiment showed that eEF2K overexpression markedly reduced the total ubiquitination level of AURKA (Figure 4I). Cycloheximide (CHX) chase experiment revealed that the degradation of AURKA was accelerated after eEF2K silencing in TNBC cells (Figure 4J). These results indicate that eEF2K interacts with and stabilizes AURKA via inhibiting its ubiquitin‐proteasome degradation.

To determine whether AURKA plays an important role in mediating the oncogenic function of eEF2K, we overexpressed eEF2K in TNBC cells with AURKA silenced (Figure S4E, Supporting Information), and showed that overexpression of eEF2K failed to promote cell proliferation, migration and invasion in TNBC cells with AURKA silenced (Figure S4F,G, Supporting Information). These results suggest that eEF2K/AURKA axis is critical in TNBC progression.

### 2.5 S391 Phosphorylation of AURKA by eEF2K is Essential for Its Oncogenic Function

**Figure 5:**
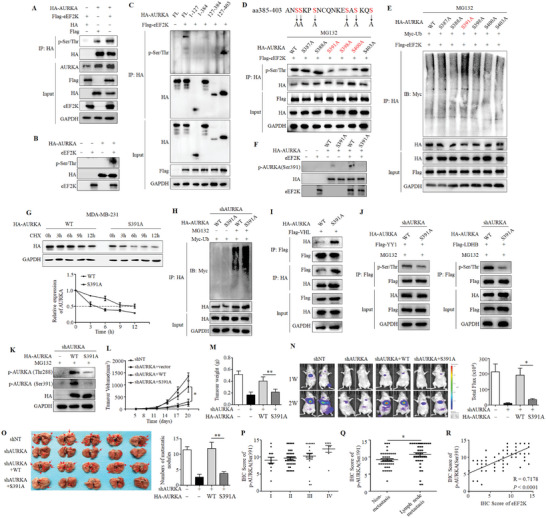
eEF2K phosphorylates AURKA at S391. A) HEK293T cells were transfected with Flag‐eEF2K or HA‐AURKA plasmids, and then subjected to immunoprecipitation with anti‐HA antibodies. The pan‐phosphorylation of AURKA was detected by western blot analysis. B) HEK293T cells were transfected with HA‐AURKA. AURKA was immunoprecipitated by anti‐HA antibody, subsequently was dephosphorylated with lambda phosphatase and then incubated with or without a recombinant active eEF2K. The reaction mixtures were subjected to western blot with anti‐pan‐serine/threonine phosphorylation antibody. C) Lysates from HEK293T cells exogenously co‐expressing Flag‐eEF2K and full‐length or different functional domains of HA‐AURKA were subjected to immunoprecipitation with the anti‐HA antibodies. The pan‐phosphorylation of different functional domains of HA‐AURKA was detected by western blot analysis. D) Lysates from HEK293T cells ectopically co‐expressing Flag‐eEF2K and wildtype or different mutants of HA‐AURKA were subjected to IP/IB with the indicated antibodies. E) HEK293T cells were exogenously co‐expressing Myc‐Ub, Flag‐eEF2K and different mutants of HA‐AURKA, followed by treatment with MG132 for 6 h. Lysates were subjected to immunoprecipitation with anti‐HA antibodies. Ubiquitination of HA‐AURKA was detected by anti‐Myc antibody. F) HEK293T cells were transfected with wildtype or S391A mutant of HA‐AURKA for 48 h. HA‐AURKA was immunoprecipitated, subsequently was dephosphorylated with lambda phosphatase and then incubated with or without a recombinant active eEF2K. The reaction mixtures were subjected to western blot with anti‐p‐AURKA (Ser391) antibody. G) MDA‐MB‐231 cells stably expressing AURKA‐targeted shRNA were transfected with ectopic wild‐type or S391A mutant AURKA, followed by treatment with CHX (10 µg ml^−1^) for the indicated time. The lysates were then subjected to western blot. H) HEK293T cells infected with AURKA shRNA lentivirus were transfected with Myc‐Ub and wild‐type or S391A mutant AURKA. HEK293T transfectants were incubated with or without MG132 for 6 h. Then, HA‐tagged AURKA was immunoprecipitated and blotted with an anti‐Myc antibody. I) HEK293T transfected with Flag‐VHL and wild‐type AURKA or S391A mutant AURKA were subjected to immunoprecipitation with anti‐Flag or anti‐HA antibodies. The reaction mixtures were subjected to western blot. J) HEK293T cells stably expressing the AURKA‐targeted shRNA were transfected with Flag‐YY1 or Flag‐LDHB and wild‐type or S391A mutant HA‐AURKA. HEK293T transfectants were incubated with MG132 for 6 h. The lysates were then subjected to immunoprecipitation with anti‐Flag antibody and western blot with an anti‐pan‐serine/threonine phosphorylation antibody. K) MDA‐MB‐231 cells stably expressing AURKA‐shRNA were transfected with empty vector, ectopic wild‐type or S391A mutant AURKA, followed by western blot analysis. L,M) MDA‐MB‐231 transfectants were subcutaneously injected into nude mice, and tumor volume and tumor weight were measured. Data are mean ± SD (n = 6). *P < 0.05, **P < 0.01. N) Representative bioluminescence images of metastasis tumors. *P < 0.05. O) Representative photographs of lung metastases were obtained from nude mice, **P < 0.01. P) Relationship between AURKA S391 phosphorylation and TNM stage. Q) The level of AURKA S391 phosphorylation in breast cancer with (n = 41) or without (n = 39) lymph node metastasis was analyzed based on immunohistochemical scores. *P < 0.05. R) Correlation between the expressions of eEF2K and AURKA S391 phosphorylation was analyzed based on immunohistochemical scores.

Since eEF2K can directly interact with AURKA (Figure 4), we asked if eEF2K affects AURKA phosphorylation. As shown in **Figure** **5**A, overexpression of eEF2K significantly increased the pan‐serine/threonine phosphorylation of AURKA. In vitro kinase assay showed that the actived‐eEF2K promoted the phosphorylation of AURKA (Figure 5B). To determine the specific phosphorylation site of AURKA by eEF2K, we co‐transfected HEK293T cells with the distinct AURKA truncations and the eEF2K expression plasmid, and found that the full‐length and aa 385–403 fragment of AURKA were phosphorylated by eEF2K (Figure 5C). Based on this, we mutated each serine and threonine residue in the aa 385–403 fragment, and found that serine phosphorylation levels of the three mutants (S391A, S398A, and S400A) were significantly reduced, as compared to the wild‐type AURKA (Figure 5D). Figure 5E showed that the ubiquitination of AURKA was significantly up‐regulated by eEF2K only when the S391 residue was mutated. In vitro kinase experiments further showed that AURKA phosphorylation was substantially downregulated by eEF2K following mutation at the S391 site (Figure S5A, Supporting Information). We then generated a specific polyclonal rabbit antibody against S391 phosphorylation of AURKA, and demonstrated that p‐AURKA (Ser391) was only observed when actived‐eEF2K was present and was decreased in the cells transfected with the AURKA S391A mutant plasmid (Figure 5F).

We next tested the effect of AURKA‐S391 on the protein stability of the kinase. Figure 5G showed that AURKA was degraded faster in the absence of its S391 phosphorylation. Additionally, the ubiquitination level of AURKA was remarkably increased in HEK293T cells transfected with AURKA S391A plasmid compared to the wild‐type plasmid (Figure 5H). Moreover, we found that there was stronger binding between AURKA and VHL, a reported E3 ubiquitin ligase of AURKA,^[^ ^25^ ^]^ in HEK293T cells transfected with AURKA S391A plasmid than that with wild‐type plasmid (Figure 5I). Together, these data suggest that S391 phosphorylation is critical for maintaining the stability of AURKA protein. Pan‐phosphorylation experiment showed that there were less phosphorylation of YY1^[^ ^26^ ^]^ and LDHB,^[^ ^27^ ^]^ as the known substrates of AURKA, in the cells transfected with AURKA‐S391A plasmid than in the cells with AURKA‐WT (Figure 5J). Re‐expression of wild‐type AURKA but not S391A mutant resulted in an increase in its phosphorylation at T288, which is required for the kinase activity of AURKA, in MDA‐MB‐231 cells with stable silencing of AURKA (Figure 5K), indicating that S391 of AURKA may structurally affect its autophosphorylation at T288. These findings suggest that S391 phosphorylation of AURKA is not only essential for its stability, but also critical for the kinase activity.

We further investigated whether S391 phosphorylation is important for the oncogenic role of AURKA. As shown in Figure S5B–D (Supporting Information), overexpression of wild type AURKA rescued the decreases of cell proliferation, migration, and invasion caused by knockdown of AURKA; AURKA S391A only had partial reversal effect. Consistently, wild type AURKA but not S391A significantly blocked the inhibition of tumor growth induced by silencing of AURKA in MDA‐MB‐231 tumor‐bearing mice (Figure 5L,M). There was no significant change in the body weights of mice (Figure S5E, Supporting Information). Additionally, wild type AURKA but not S391A increased SOX8 expression as compared to AURKA knockdown in the tumor‐bearing mice (Figure S5F,G, Supporting Information). Furthermore, overexpression of wild‐type AURKA but not S391A significantly promoted the metastasis of TNBC cells and increased the number of metastatic nodules (Figure 5N,O). Thus, S391 phosphorylation appears to be indispensable for the oncogenic function of AURKA in TNBC.

To explore the clinical implication of AURKA S391 phosphorylation in TNBC, we carried out IHC analysis on 80 TNBC specimens, and found that high level of AURKA S391 phosphorylation is correlated with advanced TNM stage of TNBC (Figure 5P). The level of AURKA S391 phosphorylation was higher in patients with the lymph node metastasis than that in those with non‐metastasis (Figure 5Q). Chi‐square test showed that AURKA S391 phosphorylation was significantly correlated with the lymph node metastasis, distant metastasis, and TNM stage but not with age, Ki67, or tumor size (Table S5, Supporting Information). There was a significant positive correlation between the expressions of eEF2K and AURKA S391 phosphorylation (Figure 5R).

We further showed that overexpression of AURKA rescued the down‐regulation of SOX8 at both protein (Figure S6A, Supporting Information) and mRNA (Figure S6B, Supporting Information) caused by eEF2K silencing in MDA‐MB‐231 cells and HCC1806 cells, implying that AURKA is an intermediate molecule in the eEF2K‐mediated regulation of SOX8 expression. Consistent with the results of in vitro experiments, knockdown of eEF2K substantially down‐regulated the protein levels of AURKA and SOX8 in the tumor‐bearing mice (Figure S6C,D, Supporting Information). The mRNA level of SOX8 was also decreased in the tumor samples with eEF2K silencing (Figure S6E, Supporting Information). Similar results were obtained with PDO samples (Figure S6F, Supporting Information). eEF2K up‐regulated the expression of SOX8 in the cells transfected with HA‐AURKA WT plasmid, but had no effect on the cells transfected with HA‐AURKA S391A plasmid (Figure S6G, Supporting Information). Furthermore, we used IHC to detect the levels of eEF2K, SOX8, and p‐AURKA (Ser391) in the primary TNBC tissues and adjacent normal breast tissues. Figure S6H (Supporting Information) showed that eEF2K, SOX8, and p‐AURKA (Ser391) were highly expressed in the primary TNBC tissues compared to the adjacent normal breast tissues, and Chi‐square test conducted on clinical samples from primary TNBC showed a significant positive correlation between eEF2K and SOX8, p‐AURKA (Ser391), as well as p‐AURKA (Ser391) and SOX8. These data demonstrated the critical role of the eEF2K/AURKA/SOX8 pathway in promoting TNBC progression.

### 2.6 Design and Synthesis of a Novel eEF2K Degrader Based on Compound C1

**Figure 6:**
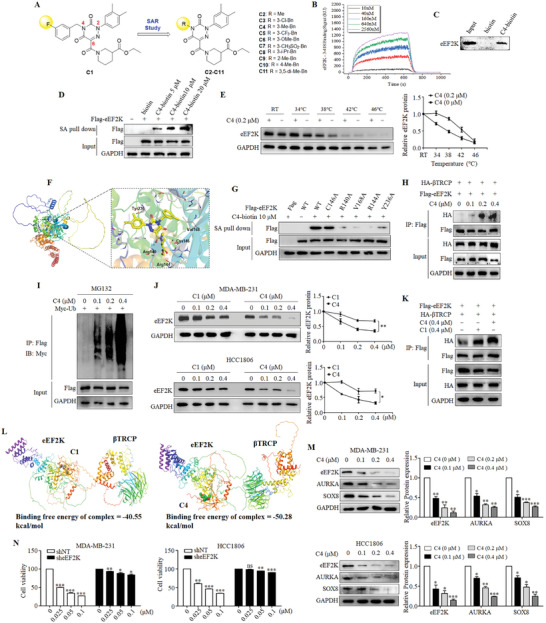
Development of a novel eEF2K degrader compound C4. A) Design and synthesis of C1 analogs based on the SAR study. B) The interaction between compound C4 and eEF2K was determined by SPR assay. C) Western blots for eEF2K protein after Biotin‐C4 pull‐down in MDA‐MB‐231 cells. D) Western blots for eEF2K protein after Biotin‐C4 pull‐down in HEK293T cells. E) Melt curves of eEF2K protein in cellular thermal shift assay (CETSA) in MDA‐MB‐231 cells treated with C4 or DMSO. F) The binding mode of C4 to the homology model of eEF2K developed by AlphaFold. C4 is shown in a yellow stick by atoms. The hydrogen bonds are labeled as dashed lines. The key amino acid residues for the binding are shown as white lines by atoms. G) Western blots for wild‐type and mutant eEF2K proteins after Biotin‐C4 pull‐down in HEK293T cells. H) HEK293T cells transfected with Flag‐eEF2K and HA‐*β*TRCP were treated with different concentrations of C4. The lysates were then subjected to immunoprecipitation with an anti‐Flag antibody. Western blot analysis was performed with the anti‐HA antibody. I) HEK293T cells transfected with Flag‐eEF2K and Myc‐Ub were treated with different concentrations of C4. Lysates were subjected to immunoprecipitation with the anti‐Flag antibodies. Ubiquitination of Flag‐eEF2K was detected by anti‐Myc antibody. J) Western blot experiment was conducted to compare the effects of C1 and C4 on eEF2K expression. **P < 0.01, *P < 0.05. K) HEK293T cells transfected with Flag‐eEF2K and HA‐*β*TRCP were treated with 0.4 µm C4 or 0.4 µm C1. The lysates were then subjected to immunoprecipitation with an anti‐Flag antibody. L) The binding free energy of 𝛽TRCP and eEF2K in the presence of C1 or C4 was analyzed by MM/GBSA calculations. M) Western blot assay was used to detect the expressions of eEF2K, AURKA, and SOX8 after treated with different concentrations of C4. ***P < 0.001, **P < 0.01, *P < 0.05. N) Effect of C4 treatment of 48 h on proliferation of stable eEF2K knockdown cell lines. The percentage of each stable cell line treated with C4 was normalized to that treated with DMSO. ns = no significance, ***P < 0.001, **P < 0.01, *P < 0.05.

We recently developed the compound C1 as a small‐molecule degrader of eEF2K and showed that this agent exhibits strong anti‐cancer activity against TNBC through down‐regulating eEF2K.^[^ ^16^ ^]^ To optimize this compound for anti‐cancer purpose, we synthesized a series of the C1 derivatives according to structure‐activity relationship (SAR) (**Figure** **6**A; Supplementary Information). As shown in Figure S7A and Table S6 (Supporting Information), compound 4 possessed the strongest anti‐cancer activity with an IC_50_ value of 24.46 nm, and had no cytotoxic effect on mammary epithelial cells MCF‐10A even at 5 µm (Figure S7B, Supporting Information). SPR binding assays showed that C4 has a strong binding affinity with eEF2K protein (Avg KD: 0.831 µm) (Figure 6B). Biotinylated C4 was demonstrated to bind to endogenous eEF2K in MDA‐MB‐231 cells (Figure 6C), and there was a stronger binding with C4 and eEF2K with the higher concentration of biotinylated C4 (Figure 6D). Cellular thermal shift assay (CETSA) verified the interaction between eEF2K and C4 (Figure 6E). These results indicate that C4 can directly bind to eEF2K. The molecular docking showed that C4 binds to the kinase active site of eEF2K in a similar manner to C1 (Figure S7C, Supporting Information). As shown in Figure 6F, the 1,2,4‐triazine‐3,5(2H,4H)‐dione scaffold of C4 also engaged in the π–π stacking interaction with residue Tyr236, and its ester group formed the hydrogen bond with residue Arg144. Despite lack of hydrogen‐bond interactions between C4 and Cys146, as previously observed in C1,^[^ ^16^ ^]^ a novel H‐bonding network was generated between the carbonyl group of the 6‐amido linkage in C4 with residue Arg140 (Figure 6F). Moreover, the 3‐methyl group on the benzyl moiety of C4 formed key van der Waals interaction with residue Val168 (Figure 6F). Consistently, eEF2K with R140A, V168A, R144A or Y236A mutants did not bind to C4, whereas eEF2K‐C146A still bound with C4 (Figure 6G). Similar to C1, C4 also enhanced the binding of beta‐transducin repeats‐containing protein  (𝛽TRCP) to eEF2K (Figure 6H), and markedly increased the ubiquitination of eEF2K (Figure 6I). QRT‐PCR results showed that C4 had no effect on eEF2K mRNA level in MDA‐MB‐231 and HCC1806 cells (Figure S7D, Supporting Information). Proteasome inhibitor MG132 could rescue the reduction of eEF2K protein caused by C4 treatment (Figure S7E, Supporting Information). Moreover, the degradation of eEF2K was accelerated in C4‐treated cells (Figure S7F, Supporting Information). Notably, C4 had a stronger effect in down‐regulating eEF2K than C1 (Figure 6J), and C4 could rapidly trigger the down‐regulation of eEF2K (within 3 h) (Figure S7G, Supporting Information). Wash‐out experiments showed that the down‐regulation of eEF2K protein was recovered more slowly in cells treated with C4 compared to C1, indicating the long‐lasting effect of C4 (Figure S7H, Supporting Information). Also, C4 had a stronger enhancing effect on the binding of eEF2K and 𝛽TRCP than C1 (Figure 6K,L). As expected, C4 significantly reduced the expressions of AURKA and SOX8 in TNBC cells (Figure 6M). As shown in Figure 6N, TNBC cells subjected to stable knock‐down of eEF2K were insensitive to C4 compared to control cells. These results suggest that C4 promotes the binding of *β*TRCP to eEF2K as a molecular glue to accelerate the degradation of eEF2K, thereby exerting anti‐cancer activity by inhibiting eEF2K‐mediating signaling pathway.

### 2.7 C4 Shows Potent Anti‐Cancer Efficacy against TNBC in vitro, in vivo, and in PDOs

**Figure 7:**
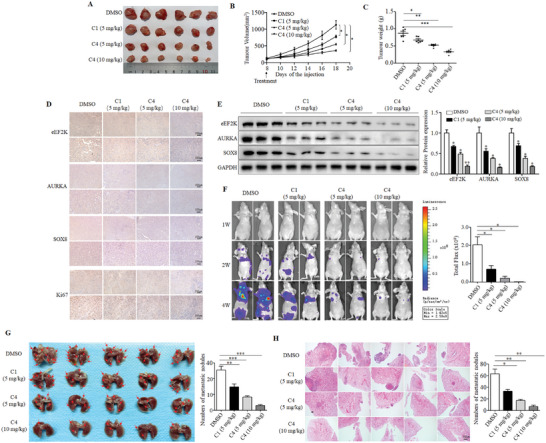
C4 exerts strong anti‐cancer activity against TNBC in vivo. Subcutaneous injection of MDA‐MB‐231 cells into 3‐week‐old female nude mice. The tumor‐bearing mice were received indicated treatment; A) Subcutaneous tumors were excised and photographs were taken at the termination of the experiment. B) Tumor sizes were measured on the days as indicated. *P < 0.05. C) Tumor weight was measured at the end of the experiments. ***P < 0.001, **P < 0.01, *P < 0.05. D) IHC assays were used to determine the expressions of eEF2K, AURKA, SOX8 and Ki67. Scale bar (top), 500 µm. Scale bar (bottom), 100 µm. E) Western blot assays were used to determine the expressions of eEF2K, SOX8, and AURKA. **P < 0.01, *P < 0.05. Three‐week‐old female nude mice were inoculated with fluorescent‐labeled MDA‐MB‐231 cells by tail vein and then received indicated treatment. F) Representative bioluminescence images of metastases tumors. *P < 0.05. G) Representative photographs of lung metastases were obtained from nude mice. ***P < 0.001, **P < 0.01. H) H&E staining of lung metastatic tumors. Scale bar, 500 µm. **P < 0.01, *P < 0.05.

**Figure 8:**
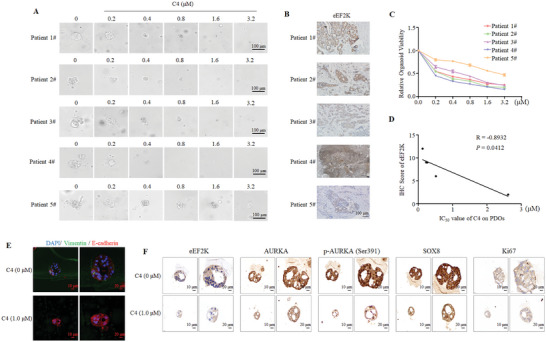
C4 exerts strong anti‐cancer activity against TNBC in PDOs. A) The morphology of organoids after C4 treatment. B) IHC detection of eEF2K expression in breast cancer patients' tissues. Scale bar, 100 µm. C) ATP‐bioluminescence assay was used to detect the viability of PDOs after C4 treated for different concentrations. D) The correlation between the expression of eEF2K and IC_50_ of C4. E) Immunofluorescence detection of vimentin and E‐cadherin expression in PDOs after C4 treatment. Scale bar (left), 10 µm. Scale bar (right), 20 µm. F) IHC detection of eEF2K, AURKA, p‐AURKA (Ser391), SOX8, and Ki67 expression in PDOs. Scale bar (left), 10 µm. Scale bar (right), 20 µm.

Next, we examined the effects of C4 on proliferation and migration of TNBC cells, and found that C4 remarkably inhibited cell viability, proliferation, migration, and invasion in a dose‐dependent manner (Figure S8, Supporting Information). Importantly, C4 triggered a stronger anti‐cancer effect as compared to C1. We further evaluated the anti‐tumor effect of C4 using a xenograft model of TNBC. The tumor volume and weight of MDA‐MB‐231 xenograft tumors were significantly decreased in C4‐treated group as compared to C1‐treated group (**Figure** **7**A–C). Consistently, the cell proliferation was significantly suppressed in the C4‐treated group, as measured by Ki67 staining of tumor sections (Figure 7D). The down‐regulations of eEF2K, AURKA, and SOX8 protein were more obvious in C4‐treated tumors than that in C1‐treated tumors (Figure 7D,E). Notably, C4 had no significant effects on body weight (Figure S9A, Supporting Information), different organ morphology (Figure S9B, Supporting Information), and hepatorenal toxicity (Figure S9C, Supporting Information). Furthermore, C4 exhibited stronger inhibitory effect on metastasis of TNBC as compared to C1, as evidenced by decreases of fluorescence signal of the lungs (Figure 7F), the number of lung metastatic nodules (Figure 7G) and H&E staining of the lungs (Figure 7H). To validate the anti‐tumor effects of C4, we test it in TNBC PDOs. We showed that the volume and budding of PDOs were significantly decreased by C4 treatment (**Figure** **8**A). PDOs with higher expression of eEF2K were more sensitive to C4 treatment, and there was a negative correlation between the IHC score of eEF2K and the IC_50_ of C4 (Figure 8B–D), indicating that the anti‐cancer effect of C4 depends on the expression of eEF2K in tumor. Immunofluorescence assays showed that C4 decreased the expression of vimentin and increased the expression of E‐cadherin in PDOs (Figure 8E). Additionally, IHC analysis demonstrated that C4 markedly suppressed the expressions of eEF2K, AURKA, p‐AURKA (Ser391) and SOX8 (Figure 8F). Also, Ki67 positive rate was significantly reduced after C4 treatment (Figure 8F).

## 3 Discussion

In this study, we found that high expression of eEF2K is associated with lymph node metastasis, distant metastasis, advanced TNM grade, and poor prognosis in TNBC patients. We further show that knockdown of eEF2K significantly inhibits TNBC growth and metastasis in vivo, in vitro and organoid experiments. Our results as well as other's studies imply eEF2K as a promising therapeutic target for TNBC.^[^ ^13^, ^28^, ^29^ ^]^ However, the molecular mechanism underlying the oncogenic role of eEF2K in TNBC remains incompletely understood.

Identification of the phosphorylation substrates of eEF2K is crucial for understanding its pro‐tumor effects. In this study, we demonstrated that eEF2K directly binds to and phosphorylates AURKA, resulting in its up‐regulation by slowing down its turnover. AURKA is a major member of the serine/threonine kinase family and plays an essential role in mitotic progression, centrosome maturation/separation, and mitotic spindle function regulation.^[^ ^30^ ^]^ In cancer, overexpression/abnormal activation of AURKA leads to aberrant regulation of tumor cell cycle, tumorigenesis/development, and cancer drug resistance in a variety of cancers, including ovarian, liver, lung, breast, and pancreatic cancers.^[^ ^31^, ^32^ ^]^ Herein, we identified a novel phosphorylation site of AURKA by eEF2K, i.e., Serine 391, and demonstrated that Serine 391 phosphorylation not only promotes the stability of AURK protein but also enhances its activity. These results suggest that AURKA‐mediated signaling pathway is involved in the regulation of eEF2K in promoting tumor progression, and the phosphorylation of AURKA by eEF2K is crucial for its function, and Serine 391 phosphorylation of AURKA may be important for the development of its inhibitor.

SOX8 has been reported to contribute to the occurrence and development of TNBC, and served as a potential molecular marker of TNBC and a promising therapeutic target.^[^ ^22^ ^]^ It has been reported that AURKA not only directly phosphorylates SOX8, but also promotes the transcription of SOX8 via regulating c‐Myc protein.^[^ ^19^ ^]^ In this study, we found that overexpression of AURKA could significantly restored the expression of SOX8 protein and mRNA in TNBC cells that were suppressed by eEF2K depletion, suggesting that the mechanism by which AURKA regulates SOX8 in ovarian cancer also exists in TNBC. Therefore, we have revealed a new mechanism for regulating the proliferation and migration of TNBC, i.e., eEF2K/AURKA/SOX8.

To date, several small molecule compounds have been reported as potential eEF2K inhibitors in anti‐cancer drug discovery, such as, 21L,^[^ ^33^ ^]^ fluoxetine,^[^ ^34^ ^]^ 18i,^[^ ^35^ ^]^ NH‐125,^[^ ^36^ ^]^ and MK‐2206.^[^ ^37^ ^]^ However, none of them exhibits comparable properties of simultaneously inhibiting eEF2K and cancer cell proliferation. The targeted protein degradation (TPD) has made spectacular progress in recent years.^[^ ^38^ ^]^ Compared to traditional small molecule protein inhibitors, which can only partially block the function of the target protein, TPD can eliminate all the functions of the target protein via degrading it.^[^ ^39^, ^40^ ^]^ Degraders can be multivalent (e.g., PROTACs) or monovalent (e.g., molecular glue degraders) depending on their modularity.^[^ ^41^, ^42^ ^]^ Recently, we reported a new compound C1, which was identified as molecular glue targeting eEF2K degradation to inhibit the carcinogenic function of eEF2K.^[^ ^16^ ^]^ Here, we successfully developed more effective eEF2K degraders through structure‐based rational design. The C1‐derived degrader C4 showed significantly improved anti‐cancer activity against TNBC proliferation and migration through in vitro, in vivo and organoid experiments. In addition, we found that C4 could effectively recruit *β*TRCP and significantly enhance the degradation of eEF2K, implying that C4 can indeed exert anti‐tumor effects as a molecular glue. It has been reported that after the protein is degraded, the molecular glue will be released to continually target other proteins, thereby rapidly reducing the level of the target protein, making it possible that a small amount of the agent can achieve a long‐lasting effect on the signaling pathway.^[^ ^43^ ^]^ Consistent with this, our results suggest that C4 has rapid (within 3 h) and long‐lasting (over 72 h) effects on the down‐regulation of eEF2K at low concentration.

## 4 Conclusion

Taken together, we report that eEF2K is a key regulator in promoting the progression of TNBC. Mechanistically, we demonstrate that eEF2K can directly interact with AURKA and phosphorylate it at the S391 site, resulting in up‐regulation of AURKA expression and its increased activity, thus increasing SOX8 expression. Importantly, we demonstrate that the newly identified phosphorylation site of AURKA, S391, is crucial for the tumor promoting effect of AURKA. The small molecule degrader C4 can markedly attenuate the malignant phenotypes of TNBC, including cell proliferation, migration, and invasion. Our study not only reveals a new signaling cascade of eEF2K to promote the progression of TNBC, but also develops a novel small molecule degrader of eEF2K, providing an effective therapeutic strategy for treatment of TNBC.

## 5 Experimental Section

### Clinical Samples

A total of 64 fresh frozen paired samples of primary breast cancer and adjacent normal breast tissue were collected from the Second Xiangya Hospital of Central South University (Changsha, China). 80 paraffin‐embedded samples of primary TNBC were obtained from the Department of Pathology at the Second Xiangya Hospital.

### PDOs

Tissue samples from human TNBC patients were subjected to organoid culture experiments within 2 h. In brief, after being received, the tissue samples were cut up and digested into single‐cell suspensions, which were then cultured in a 3D environment constructed with 50% cold Matrigel (Corning, NY, USA). After being cultured for 5 days, the organoids were collected for subsequent experiments. For lentivirus infection, the organoids were digested into single‐cell suspension with TrypLE Express (Invitrogen, Carlsbad, CA, USA), and then incubated with the lentivirus for 24 h. The infected cells were planted in 96‐well plates with 1000 cells per well, and cell morphology was observed and cell activity was detected after 5 days. For drug sensitivity detection, the organoids were digested into single‐cell suspension, planted in 384‐well plates (2000 cells/well), and treated with indicated compounds. After 4 days of treatment, cell viability was measured using CellTiter‐Glo 3D Reagent (Promega, Beijing, China), following the manufacturer's protocol.

### Xenograft Model

BALB/c nude mice were used for animal studies. The mice were randomly assigned and treated in an unblinded manner. 2 × 10^6^ MDA‐MB‐231 stable cell lines were subcutaneously injected into the right flank of the mice in each group. To assess the in vivo effect of compounds, BALB/c nude mice were randomly divided into indicated groups and subcutaneously injected with 2 × 10^6^ MDA‐MB‐231 cells. After eight days of growth, the indicated compounds were injected intraperitoneally every other day. Tumor growth was regularly monitored with the tumor size measured with calipers every 2 days. The tumor volume was calculated as length × width^2^ × (π/6). At the termination of the experiment, the tumors were removed, photographed, and weighed.

### In Vivo Fluorescence Imaging

The in vivo metastasis experiment was performed on 3‐week‐old female BALB/c nude mice. The mice were randomly assigned and treated in an unblinded manner. The metastasis model was established by injection of 2 × 10^5^ MDA‐MB‐231 cells resuspended in 50 µL of PBS. To assess the antimetastatic effects of the indicated compounds, the mice were treated with the indicated compounds by intraperitoneal injection every 2 days. Fluorescence signals were tracked weekly with a PerkinElmer in vivo optical imaging system (IVIS Lumina). Images were analyzed with Living Imaging Software. At the end of the experiment, the mice were euthanized, the lungs were harvested, and the number of metastatic nodules was observed under an anatomical microscope.

### Reagents and Antibodies

Antibodies used in immunoblotting were as follows: anti‐eEF2K (ab45168, ab85721, 1:1000) and p‐Ser/Thr eEF2K (ab17464, 1:1000) were purchased from Abcam (Cambridge, England), and anti‐AURKA (No. 14475S, 1:1000) and anti‐AURKA Thr288 (No. 3079S, 1:1000) were purchased from Cell Signaling Technology (Boston, MA, USA). Anti‐GST (10000‐0‐AP, 1:4000), anti‐Flag (66008‐3‐lg, 1:5000), anti‐SOX8 (20627‐1‐AP, 1:1000), and anti‐HA (81290‐1‐RR, 1:1000) antibodies were purchased from Proteintech (Wuhan, China). Anti‐Myc (100029‐MM08, 1:5000) was purchased from Sino Biological Inc. (Beijing, China). Anti‐E‐cadherin (EM0502, 1:2000), anti‐N‐cadherin (ET1607‐37,1:2000), and anti‐vimentin (ET1610‐39, 1:2000) were purchased from HUABIO (Hangzhou, China). Anti‐GAPDH (GB15002‐100, 1:2000) was purchased from Servicebio (Wuhan, China). The specific polyclonal rabbit antibody against the S391 phosphorylation site of AURKA was generated by ABclonal Technology (Wuhan, China). Normal IgG/peroxidase‐conjugated AffiniPure Goat Anti‐rabbit/mouse IgG (H+L) was purchased from the Jackson ImmunoResearch (West Grove, PA, USA). The proteasome inhibitor MG132 was purchased from MedChem Express (Shanghai, China). The protein synthesis inhibitor CHX was purchased from TargetMol (Shanghai, China).

### Cell Line and Culture

The human breast cancer cell line MDA‐MB‐231 and the human embryonic kidney cell line HEK293T were cultured in Dulbecco's Modified Eagle Medium (Gibco, Carlsbad, CA, USA). The human breast cancer cell line HCC1806 was cultured in RPMI 1640 medium (Gibco). All the cell culture media used were supplemented with 10% fetal bovine serum (ExCell Bio, Suzhou, Jiangsu, China), along with streptomycin (100 µg/mL) and penicillin (100 U/mL). The normal mammary epithelial cell line MCF‐10A was cultured in specific epithelial culture medium (Procell Life Science & Technology Co., Ltd. Wuhan, China). All the cell lines were identified by STR method and cultured at 37 °C in a humid atmosphere containing 5% CO_2_/95% air.

### EdU Assay

According to the manufacturer's instructions, cells subjected to different treatments were incubated with 50 µM 5‐Ethynyl‐2′‐deoxyuridine assay (EdU, Ribobio, Guangzhou, China) for 2 h at 37 °C followed by fixation with 4% paraformaldehyde at room temperature for 20 min. The fixed cells were then treated with 2 mg/ml glycine for 5 min, followed by permeation with 0.5% Triton X‐100 for 30 min. Next, the cells were stained with a 1× Apollo reaction cocktail for 30 min and exposed to Hoechst 33342 (5 µg/ml) for 30 min at room temperature. Finally, the cells were observed and imaged under a fluorescence microscope.

### Wound Healing Assay

Cells were inoculated in 12‐well plates and cultured in serum‐free medium. When the cell density reached ≈90%, the cells were scratched with the tip of a yellow pipette. The wound area was measured by ImageJ software at the indicated time points and normalized by the initial time point

### Immunohistochemical Staining

Paraffin‐embedded breast cancer tissue specimens were sectioned at a thickness of 4 µm. After deparaffinization in xylene and rehydration with gradients of alcohol, the slides were immersed in 0.01 mol/L citric acid buffer (pH 6.0) and heated at 95 °C for 15 min for antigen retrieval. Next, the slides were incubated with 3% hydrogen peroxide (H_2_O_2_) for 10 min, blocked with 10% normal goat serum for 10 min, and incubated with the indicated antibodies at room temperature for 1 h. After being washed with PBS containing Tween 20 three times, the slides were incubated with a biotin‐labeled secondary antibody and horseradish peroxidase (HRP)‐conjugated streptavidin for 30 min at room temperature. Subsequently, the slides were soaked in 0.01% H_2_O_2_ with HRP substrate and 3.3′‐diaminobenidine tetrahydrochloric acid (Sigma, St. Louis, MO, USA) for 10 min and counterstained with Meyer's hematoxylin for 30–60 s. Finally, the slides were mounted with mounting medium for visualization under a microscope. Protein expression levels were scored independently by two experienced pathologists using a previously published method.

### Immunofluorescence Staining

MDA‐MB‐231 cells were seeded in confocal plates. After being cultured for 24 h, cells were fixed with 4% paraformaldehyde for 20 min at room temperature, blocked in 1% bovine serum albumin (BSA) for 1 h, and incubated with anti‐eEF2K and anti‐AURKA antibodies at 4 °C overnight. The cells were then incubated with Alexa Fluor 594 dye‐coupled anti‐rabbit IgG antibody and Alexa Fluor 488 dye‐coupled anti‐mouse IgG antibody. Finally, cells were stained with 4, 6‐diamino‐2‐phenylindole (DAPI) and the fluorescence signal was visualized by confocal microscopy.

### Western Blot Assay

Cells were lysed on ice for 30 min in RIPA lysis buffer (Boster, Beijing, China) containing protease and phosphatase inhibitors, and proteins were collected after centrifugation at 12 000 rpm for 15 min. For western blot analysis, equal amounts of proteins (30 µg) were separated on SDS‐PAGE gel and transferred to ImmobilonPVDF membrane (Merck KGaA, Darmstadt, Germany). 5% non‐fat milk was used for blocking for 1.5 h at room temperature. Then, the PVDF membranes were incubated with the indicated primary antibodies at 4 °C overnight followed by incubation with anti‐mouse or rabbit secondary antibodies for 1 h at room temperature. Finally, the immune complex signals were visualized using the enhanced chemiluminescence (ECL) (US EVERBRIGHT, Suzhou, China).

### Co‐Immunoprecipitation Assay

Cells were lysed with RIPA lysis buffer (medium) containing protease inhibitors and phosphatase inhibitors, and protein complexes were obtained after centrifugation at 12 000 rpm for 15 min, followed by incubation with indicated primary antibodies and protein A/G magnetic beads overnight at 4 °C. Protein A/G magnetic beads were adsorbed with a magnetic rack and washed three times with PBS. 2X sodium dodecyl sulfate‐polyacrylamide gel electrophoresis (SDS‐PAGE) sample buffer (New Cell & Molecular Biotech, Suzhou, China) was added to the protein A/G magnetic beads and boiled for 10 min, followed by western blot analysis.

### RNA Extraction, Reverse Transcription, and qRT‐PCR

For RNA extraction, cells were treated with TRIzol reagent (CWBio, Taizhou, China) and the total RNA was extracted with trichloromethane. Next, the extracted RNA was washed with isopropyl alcohol and 75% ethanol. For reverse transcription, complementary DNA (cDNA) was synthesized from 1 µg of total RNA using the PrimeScript RT Kit (TaKaRa, Japan). QRT‐PCR was performed on a qRT‐PCR machine (Life Technologies, USA) and analyzed using the QuantStudio Design & Analysis Software v1.5.1. The relative RNA abundance was calculated using the standard 2^−ΔΔCt^ method. The primers used are listed in Table S1 (Supporting Information).

### siRNA, shRNA, and Plasmid Transfection

The siRNAs used in this study were purchased from RiboBio, and siRNA transfection was performed according to the manufacturer's protocol. Briefly, cells in the exponential growth phase were plated in 6‐well culture plates. When the cell density reached ≈40%, Lipofectamine RNAiMAX reagent and Opti‐MEM‐reduced serum medium were used for siRNA transfection. Cells with stable knockdown of indicated genes were constructed by lentivirus‐based shRNA infection and selected by adding 1 µg/mL puromycin to the culture medium. Plasmid transfection was performed with Lipofectamine 8000 (Beyotime Biotechnology, Shanghai, China) reagent according to the manufacturer's protocol. The target sequences of the siRNAs and shRNAs are listed in Table S2 (Supporting Information).

### Clonogenic Assay

800 cells/well were seeded in 6‐well culture plates and then cultured in an incubator for 14 days. Then, the cells were fixed with 4% paraformaldehyde and stained with crystal violet for 20 min. Finally, the colonies were counted. The designated drug was added to the cells subjected to drug treatment on the second day of seeding and the culture medium containing the drug was changed every 72 h.

### Cell Counting Kit 8 (CCK‐8) Assay

Cell viability was measured by a CCK‐8 assay. In brief, cells were planted in 96‐well culture plates in a number of 2500 cells per well and subjected to different treatments in specified time. For cell viability assessment, 10 µL CCK‐8 reagent diluted in 90 µL culture medium was added to each well and incubated in an incubator for 1.5 h. The OD value at 450 nm was determined.

### GST Pulldown Assay

The HA‐AURKA plasmid was transfected into HEK293T cells for 48 h. Obtained cell lysate was incubated with purified GST or GST‐eEF2K (Sigma) at 4 °C for 4 h. The binding protein was detected by western blot analysis.

### In Vitro Kinase Assay

The HA‐AURKA wild type or different mutants of HA‐AURKA plasmids were transfected into HEK293T cells. After 48 h, HA‐AURKA was immunoprecipitated with anti‐HA antibody and protein A/G magnetic beads, and pretreated with lambda phosphatase. Briefly, the immunoprecipitation mixture was reacted with 1X lambda phosphatase buffer, 1X MnCl2 buffer, and 20 U/µl lambda protein phosphatase at 30 °C for 30 min, and then the protein A/G magnetic beads were washed three times with PBS buffer. For the in vitro kinase assay, the cleaned protein A/G magnetic beads were incubated with 200 ng of active eEF2K in 1× kinase buffer (Cell Signaling Technology). ATP at final concentration of 200 µM was added (Cell Signaling Technology). After incubation at 30 °C for 1 h, the reaction was terminated by adding SDS‐PAGE loading buffer, and western blot analysis was performed.

### CETSA

MDA‐MB‐231 cells were pretreated with the compound or DMSO for 30 min, and the cells were harvested and washed three times with PBS. The cells were then suspended in PBS containing protease inhibitors and phosphatase inhibitors and maintained with the same dose of compound or DMSO as in the initial treatment. The cell suspension was divided into 7–10 centrifuge tubes with 100 µl volume and each tube was designated a temperature point. After heating at designated temperature for 2 min, the tube was removed and incubated at room temperature for 3 min. Samples were immediately snap‐frozen in liquid nitrogen, and stored at ‐80 °C. The cells were centrifuged at 20 000 g for 20 min at 4 °C to remove cell debris, and the collected proteins were used for western blot experiments.

### The Synthesis of C1 Analogues

Reagents and conditions: (a) K_2_CO_3_, substituted benzyl bromide or benzyl bromide, acetonitrile, r.t, overnight, 74–85%. (b) i) Concentrated HCl, AcOH, 110 °C, 70–80%; ii) DCM, oxalyl chloride, N_2_, 0 °C, overnight; iii) R^′^H, DCM, r.t, overnight, 30–90%. The specific method is described in the supplementary information.

### Molecular Docking

The full‐length human protein structures of eEF2K (AF‐O00418‐F1‐model_v3.pdb) and *β*TRCP (AF‐Q9Y297‐F1‐model_v3.pdb) were obtained from the AlphaFold Protein Structure Database.^[^ ^44^, ^45^ ^]^ For each homology model, all water molecules were removed, damaged side chains were repaired, and missing hydrogen atoms were added using the *Protein Preparation Wizard* module of Schrödinger 9.0. Protonation states and partial charges were assigned using the OPLS2005 force field. Subsequently, the *Receptor Grid Generation* module was utilized to define the binding pocket, which was set to a size of 20 × 20 × 20 Å and centered on the centroid of three key residues including Lys170, Glu229 and Tyr236 previously reported as the active site of eEF2K.^[^ ^16^ ^]^ Compound C4 was preprocessed using the *LigPrep* module, generating tautomers at pH = 7.0 ± 2.0 and producing various combinations of chiralities by setting the maximum number of stereoisomers in *Epik* to 32. Using the extra precision (XP) scoring function of Glide docking, compound C4 was docked into the binding pocket of eEF2K, and the docking pose and binding affinity were predicted and evaluated. Finally, the interaction patterns and binding affinities between eEF2K and *β*TRCP, as well as the binding complex formed by compound C4 with eEF2K and *β*TRCP, were assessed using the HawkDock service.^[^ ^46^, ^47^ ^]^

### Statistical Analysis

GraphPad Prism 6.0 was used for statistical analysis. Chi‐squared test and one‐way ANOVA were used to compare the differences in clinical variables among groups in the TNBC cohorts. Student's t‐test was used to analyze two group comparisons. One‐way analysis of variance was used to compare multiple groups (> 2). Kaplan‐Meier survival analyses were used to evaluate the prognostic significance of eEF2K levels in TNBC patients. All the experiments were performed in triplicate. The results are shown as the mean ± SD. *P* values < 0.05 were considered statistically significant.

## Conflict of Interest

The authors declare no conflict of interest.

## Author Contributions

X.W. and R.G. contributed equally to this work. Y.C., N.Y., and J.Y. designed the study and revised the manuscript. X.W. and R.G. performed the experiments, analyzed the experimental data, and drafted the manuscript. X.Z. and R.Z. designed and synthesized C1 derivatives. S.T. conducted molecular docking experiments. C.Z. and S.C. performed pharmacological experiments. W.Y. and Z.C. collected clinical samples, performed immunohistochemistry, and analyzed the results of immunohistochemistry. Y.L. and T.S. helped with data analysis. S.J. and L.H. provided technical support in the experiments.

## Supporting information



Supporting Information

## Data Availability

All data generated or analyzed during this study are included in this article and its supplementary files.
