# Supplementary material for: Identification of a Novel Substrate for eEF2K and the AURKA‐SOX8 as the Related Pathway in TNBC
Source: Adv Sci (Weinh). 2025 Feb 14;12(14):2412985. doi: 10.1002/advs.202412985 (PMC11984844; doi:10.1002/advs.202412985)
Supplement: Supplementary file 1 — Supporting Information [file ADVS-12-2412985-s001.docx]

**Supplementary Materials for**

**Identification of a novel substrate for eEF2K and the AURKA-SOX8 as the related pathway in TNBC**

Xiaoya Wan et al.

Corresponding author: Yan Cheng, yancheng@csu.edu.cn

The PDF file includes:

Supplementary Figure 1 to 9;

Supplementary Table 1 to 6.


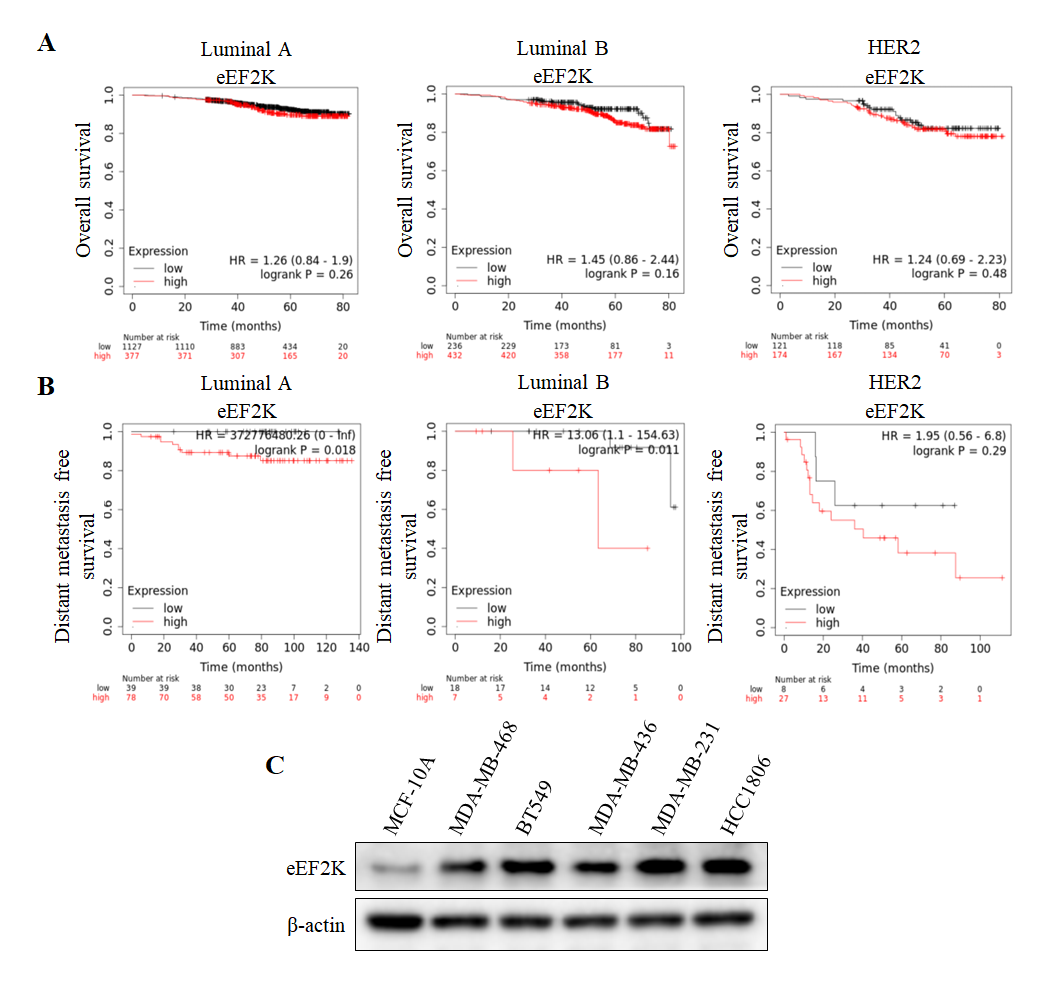


**Figure S1. The correlation between eEF2K expression and prognosis in different subtypes of breast cancer.**

**(A-B)** The correlation between eEF2K expression and overall survival (OS) (A) or distant metastasis free survival (DMFS) (B) in breast cancer patients with luminal A, luminal B, and human epidermal growth factor receptor 2 (HER2) subtypes was analyzed by online Kaplan-Meier plotter website. **(C)** The eEF2K expression level in normal mammary epithelial cell line and five TNBC cell lines were determined by western blot.


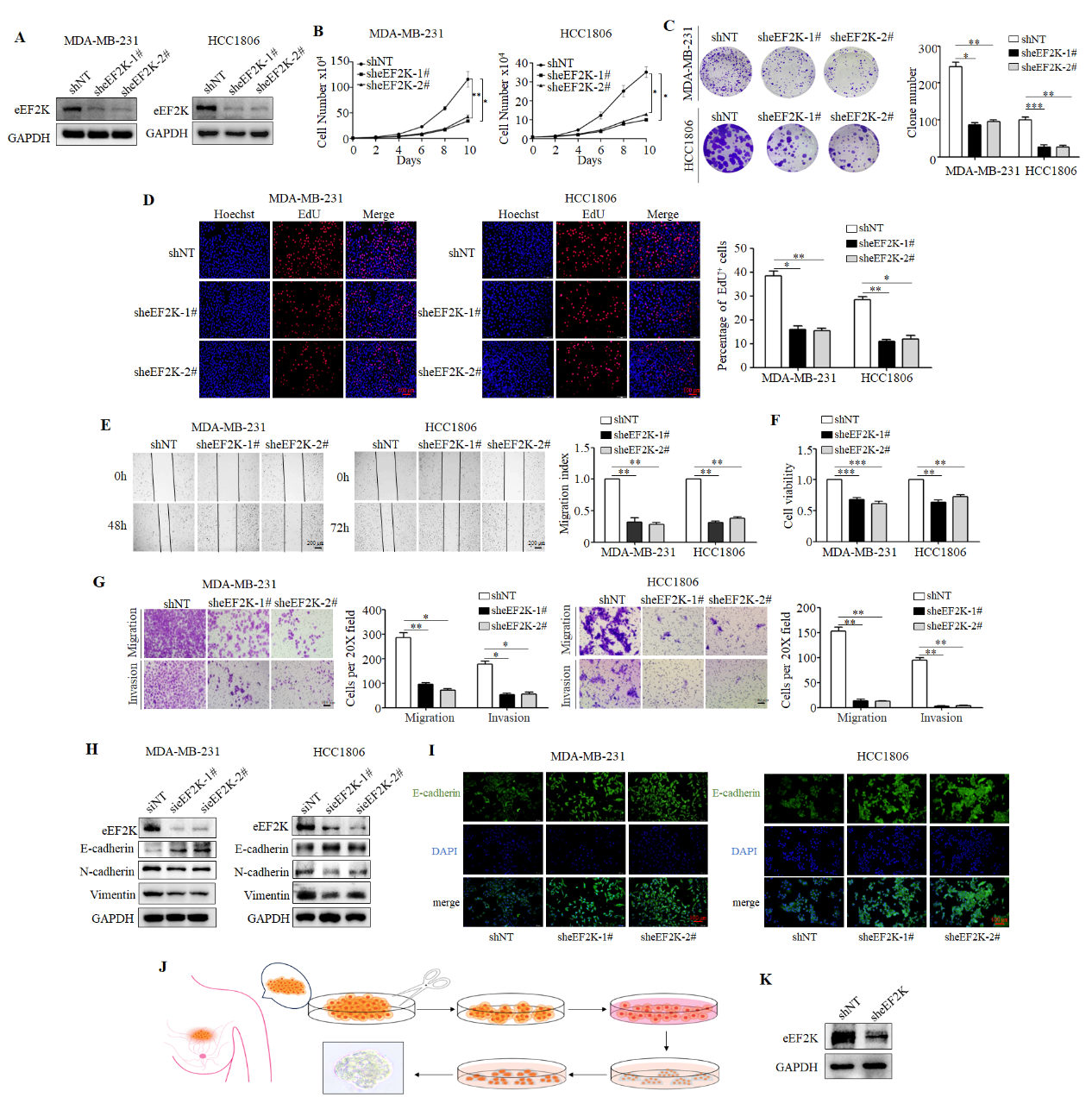


**Figure S2. Silencing of eEF2K inhibits the progression of TNBC**

**(A)** MDA-MB-231 and HCC1806 cells were transfected with eEF2K shRNA. The expressions of eEF2K were measured by western blotting. **(B)** The growth rate of eEF2K-knockdown MDA-MB-231 and HCC1806 cells was determined by cell number assay. ***P <* 0.01, **P <* 0.05. **(C)** The inhibition of cell proliferation induced by eEF2K knockdown was confirmed by colony formation assay. ****P <* 0.001, ***P <* 0.01, **P <* 0.05. **(D)** EdU staining assays were conducted to assess the proliferation ability of TNBC cells. Scale bar, 100 μm. ***P <* 0.01, **P <* 0.05. **(E)** Wound healing assays were used to assess cell migration ability following eEF2K silencing. Scale bar, 200 μm. ***P* < 0.01. **(F)** CCK8 assays were used to detect the proliferation of eEF2K knockdown cells under the same conditions as the wound healing assay. ****P* < 0.001, ***P* < 0.01. **(G)** Transwell assays were performed to evaluate the migration and invasion abilities of MDA-MB-231 and HCC1806 cells. Scale bar, 100 μm. ***P* < 0.01, **P* < 0.05. **(H)** The expressions of N-cadherin, vimentin and E-cadherin after silencing eEF2K were detected by western blot assay. **(I)** Immunofluorescence assays were used to determine the expression of E-cadherin. Scale bar, 100 μm. **(J)** Schematic diagram of PDOs culture. **(K)** Western blot assay was used to detect the expression of eEF2K in PDO after silencing eEF2K.


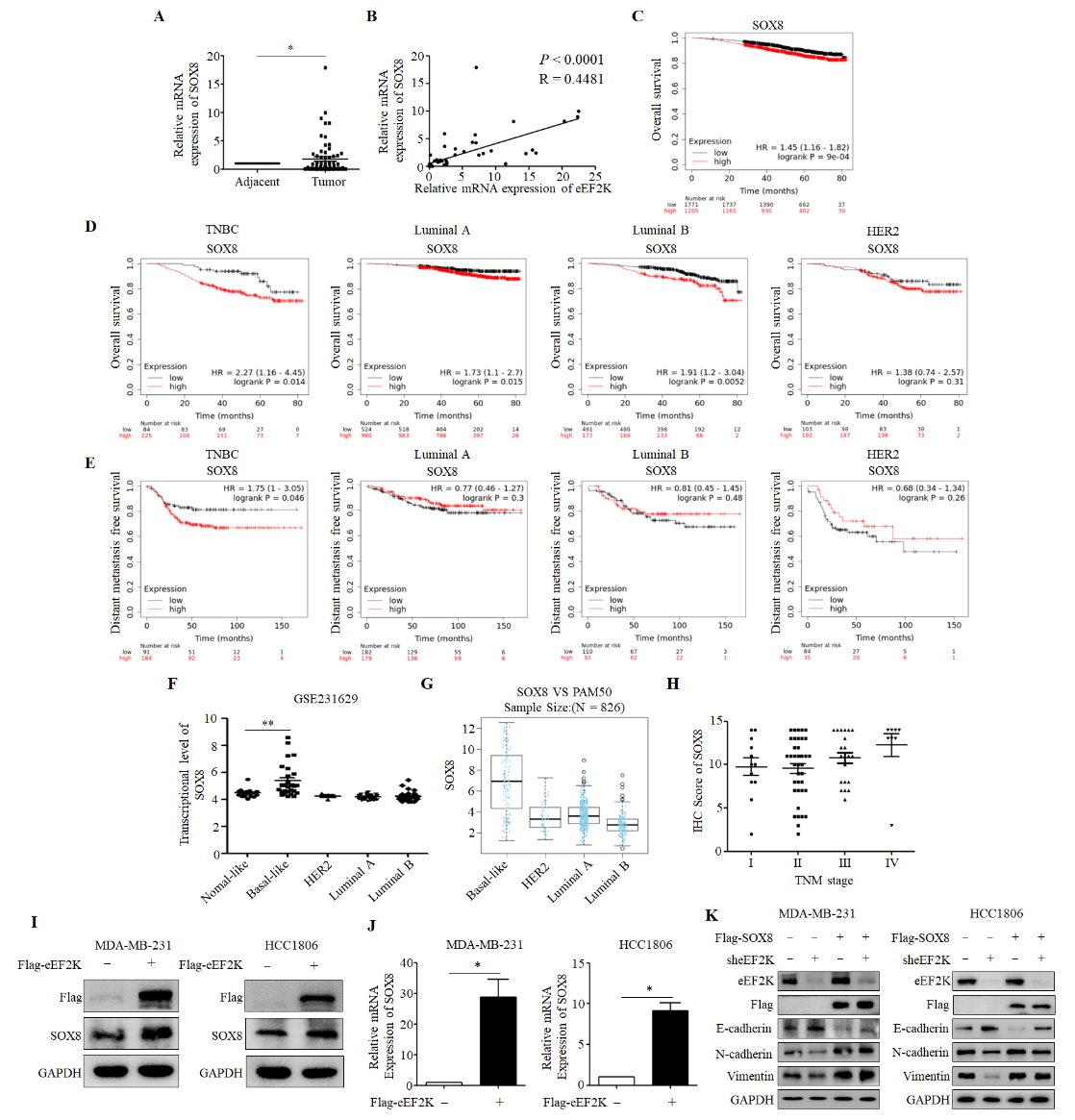


**Figure S3. SOX8 promotes the malignant phenotype of TNBC**

**(A)** The SOX8 mRNA expression in 64 paired breast cancer tissues and adjacent non-tumor tissues were measured by qRT-PCR. GAPDH was the internal control. **P* < 0.05. **(B)** The correlation between mRNA expressions of eEF2K and SOX8 was analyzed based on 64 breast cancer tissues. **(C)** Online Kaplan-Meier plotter website analyzes the correlation between SOX8 expression and OS in breast cancer patients. **(D-E)** Online Kaplan-Meier plotter website analyzed the association between SOX8 expression and OS (D) or DMFS (E) in patients with different subtypes of breast cancer. **(F****)** The expression of SOX8 in different breast cancer subtypes was analyzed using GSE231629 breast cancer cohorts. ***P <* 0.01. **(G)** The expression of SOX8 in different breast cancer subtypes were analyzed using LinkedOmic database. **(H)** The relationship between SOX8 expression and TNM stage. **(I)** Western blot assay was used to detect the protein expression of SOX8 upon overexpression of eEF2K. **(J)** The mRNA expression of SOX8 upon overexpression of eEF2K was measured by qRT-PCR. GAPDH was the internal control. **P <* 0.05. **(K)** MDA-MB-231 and HCC1806 cells stably expressing eEF2K shRNA and the non-target control were transfected with Flag-SOX8 plasmid. The expressions of E-cadherin, vimentin and N-cadherin were measured by western blot.


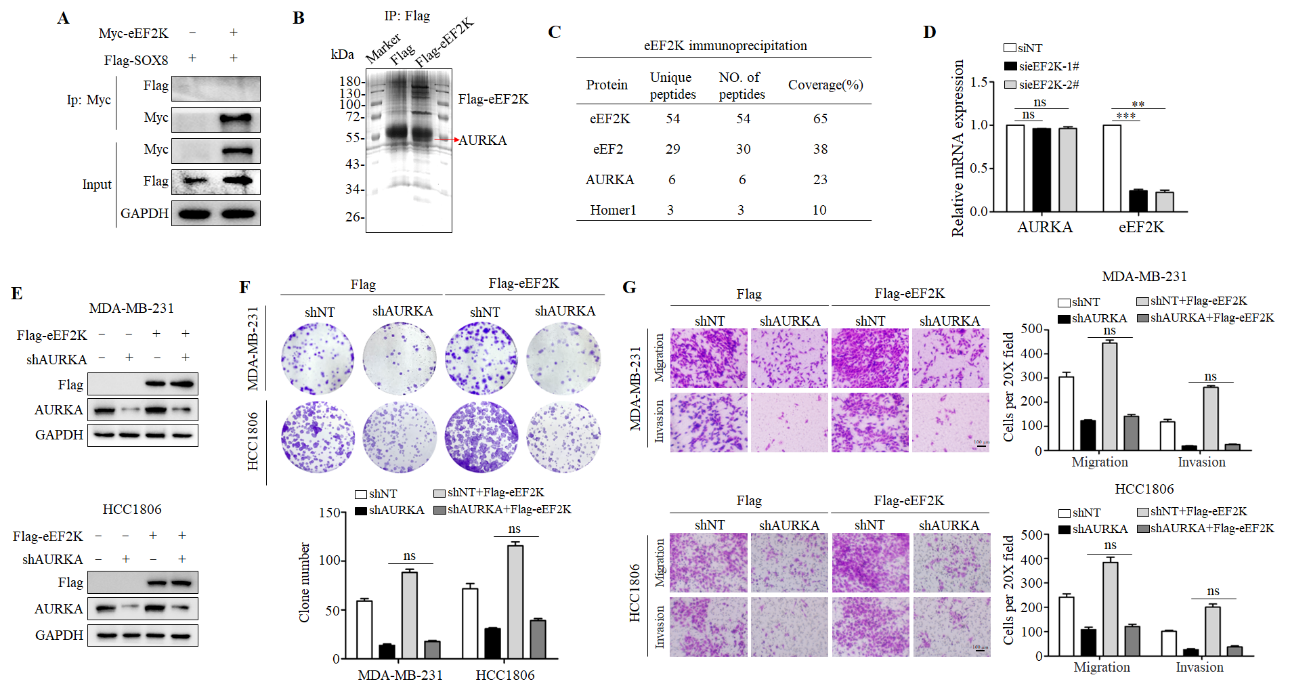


**Figure S4.** **AURKA but not SOX8 interacts with eEF2K**

**(A)** HEK293T cells were transfected with Myc-eEF2K and Flag-SOX8 plasmids, and then subjected to immunoprecipitation with anti-Myc antibody. The lysates and immunoprecipitates were then blotted. **(B)** Proteins from the immunoprecipitates assay were separated by polyacrylamide gel eletrophoresis and detected by coomassie staining. The proteins immunoprecipitated by anti-Flag antibody were further analyzed by mass spectrometry. **(C)** Representative proteins that interact with eEF2K. **(D)** The mRNA expression level of AURKA was detected by qRT-PCR after eEF2K was downregulated. ns, no significance. ****P* < 0.001, ***P* < 0.01. **(E)** MDA-MB-231 and HCC1806 cells stably expressing the AURKA-targeted shRNA and the non-target control were transfected with Flag-eEF2K expression plasmid. Western blot assay was used to detect the protein expression of AURKA and Flag-eEF2K. **(F)** Colony formation assays were conducted to assess the proliferation ability of TNBC cells. ns, no significance. **(G)** Transwell assays were performed to evaluate the migration and invasion abilities of MDA-MB-231 and HCC1806 cells. Scale bar, 100 μm. ns, no significance.


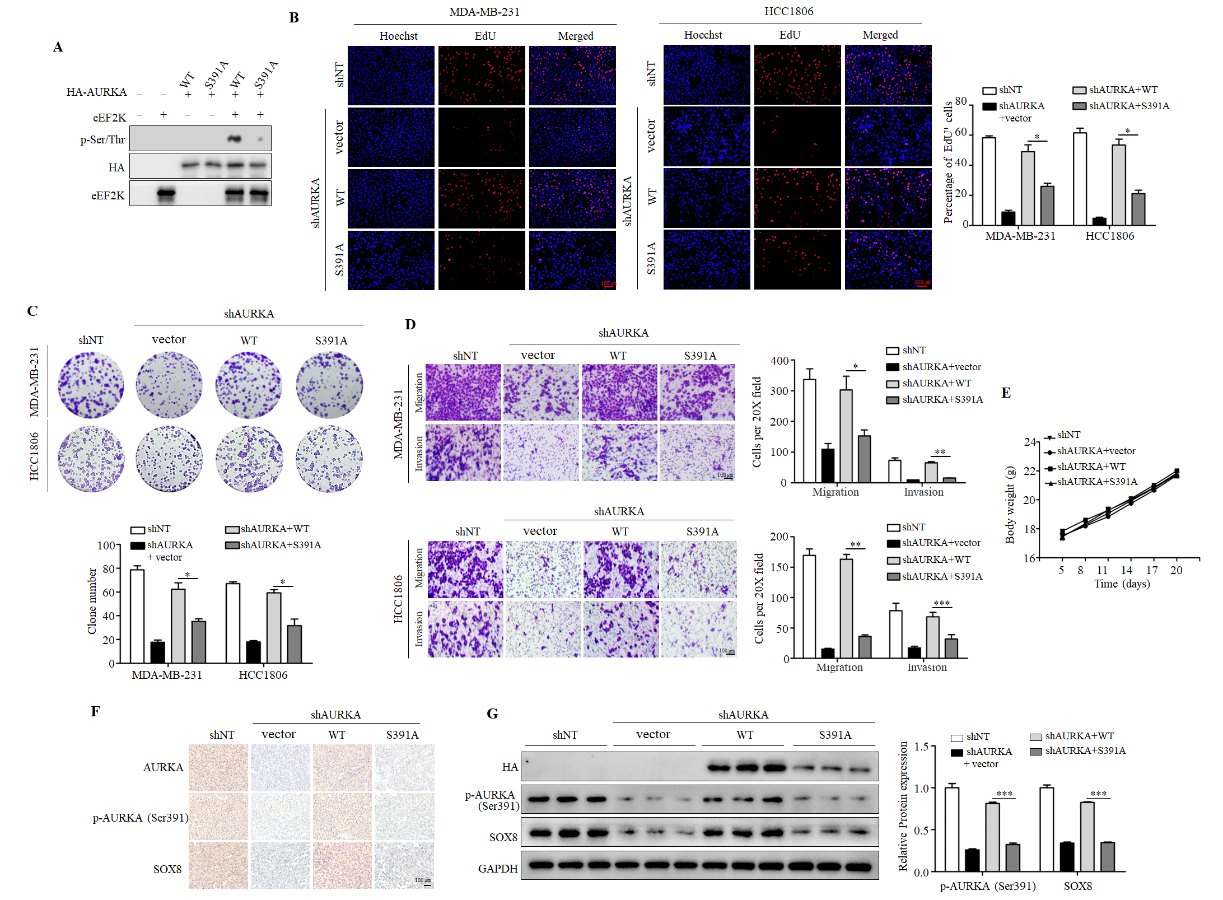


**Figure S5. S391 phosphorylation is important for the oncogenic role of AURKA.**

**(A)** HEK293T cells were transfected with wildtype or S391A mutant of HA-AURKA for 48 h. HA-AURKA was immunoprecipitated, subsequently was dephosphorylated with lambda phosphatase and then incubated with or without a recombinant active eEF2K. The reaction mixtures were subjected to western blot with anti-pan-serine/threonine phosphorylation antibody. MDA-MB-231 and HCC1806 cells stably expressing AURKA shRNA were transfected with empty vector, ectopic wild-type and S391A mutant AURKA, respectively; **(B)** EdU staining assays were conducted to assess the proliferation ability. Scale bar, 100 μm. ***P* < 0.01, **P* < 0.05; **(C)** Colony formation assays were applied to evaluate cell proliferation ability. ***P* < 0.01, **P* < 0.05; **(D)** Transwell assays were performed to evaluate the migration and invasion abilities. Scale bar, 100 μm. ****P* < 0.001, ***P* < 0.01, **P* < 0.05. **(E)** MDA-MB-231 transfectants were subcutaneously injected into nude mice, and body weight was measured. **(F)** IHC was performed to determine the protein levels of AURKA, p-AURKA (Ser391), and SOX8 in xenograft tumors. Scale bar, 100 μm. **(G)** The protein expressions of AURKA, p-AURKA (Ser391), and SOX8 in xenograft tumors were detected by western blot. ****P* < 0.001.


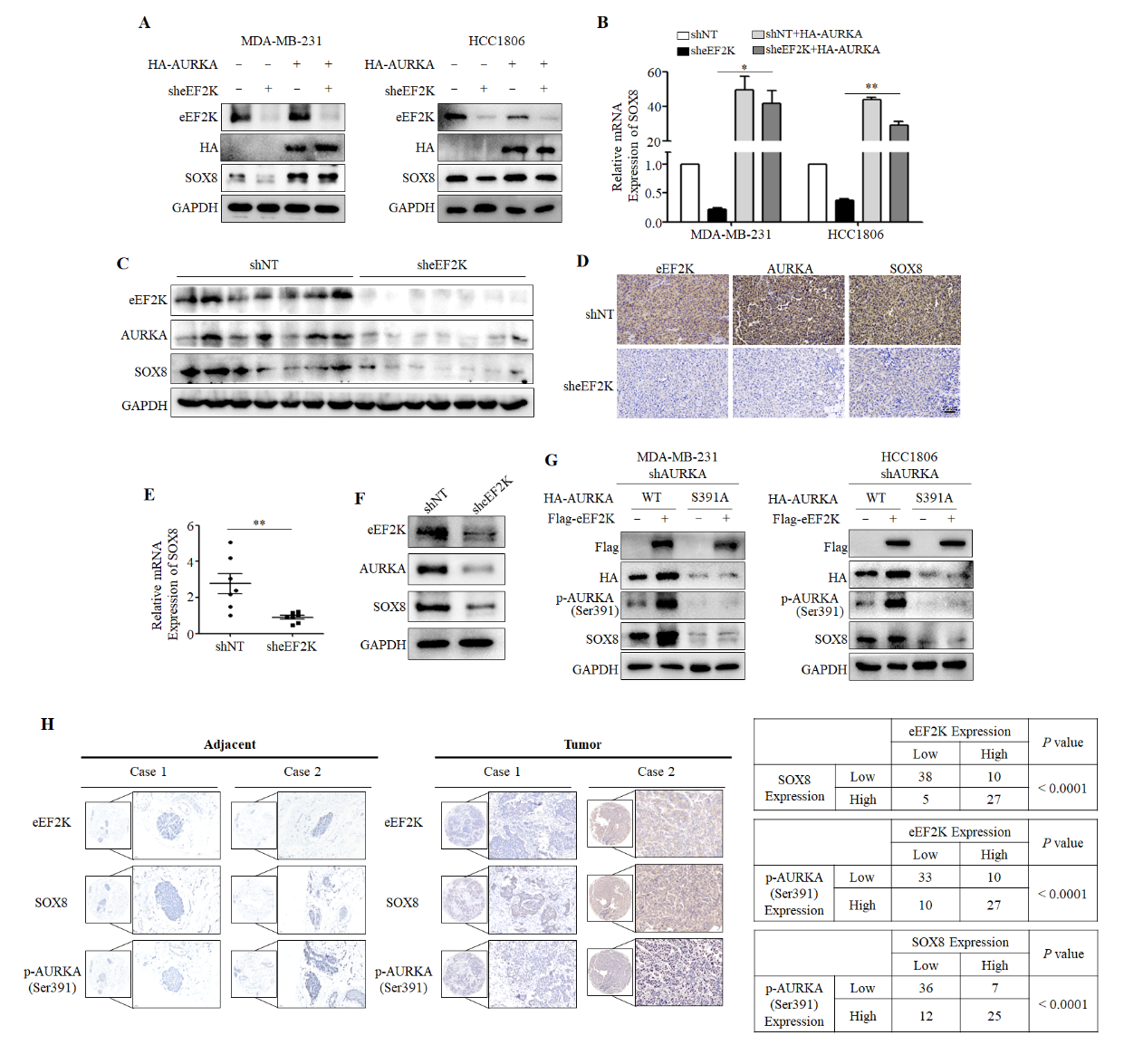


**Figure S6. AURKA mediates the up-regulation of SOX8 induced by eEF2K**

MDA-MB-231 and HCC1806 cells stably expressing eEF2K shRNA and the non-target control were transfected with HA-AURKA expression plasmid. **(A)** The protein expression of SOX8 was determined by western blot. **(B)** The mRNA expression of SOX8 was determined by qRT-PCR. GAPDH was the internal control. ***P* < 0.01, **P* < 0.05. **(C)** The protein expressions of eEF2K, AURKA, and SOX8 in xenograft tumors were detected by western blot. **(D)** IHC was performed to determine the protein levels of eEF2K, AURKA, and SOX8 in xenograft tumors. Scale bar, 50 μm. **(E)** The mRNA expression of SOX8 in xenograft tumors was detected by qRT-PCR. ***P* < 0.01. **(F)** The protein expression of eEF2K, AURKA and SOX8 in PDO with eEF2K silencing were detected by western blot. **(G)** MDA-MB-231 and HCC1806 cells stably expressing AURKA shRNA were transfected with wildtype, S391A mutant of HA-AURKA or Flag-eEF2K plasmid. p-AURKA (Ser391), HA-AURKA and SOX8 expressions were detected by western blot. **(H)** Representative images of eEF2K, SOX8, and p-AURKA (Ser391) expression in primary TNBC and adjacent normal breast tissue. The chi-square test was used to analyze the correlation between eEF2K, p-AURKA (Ser391), and SOX8 in clinical samples of primary TNBC.


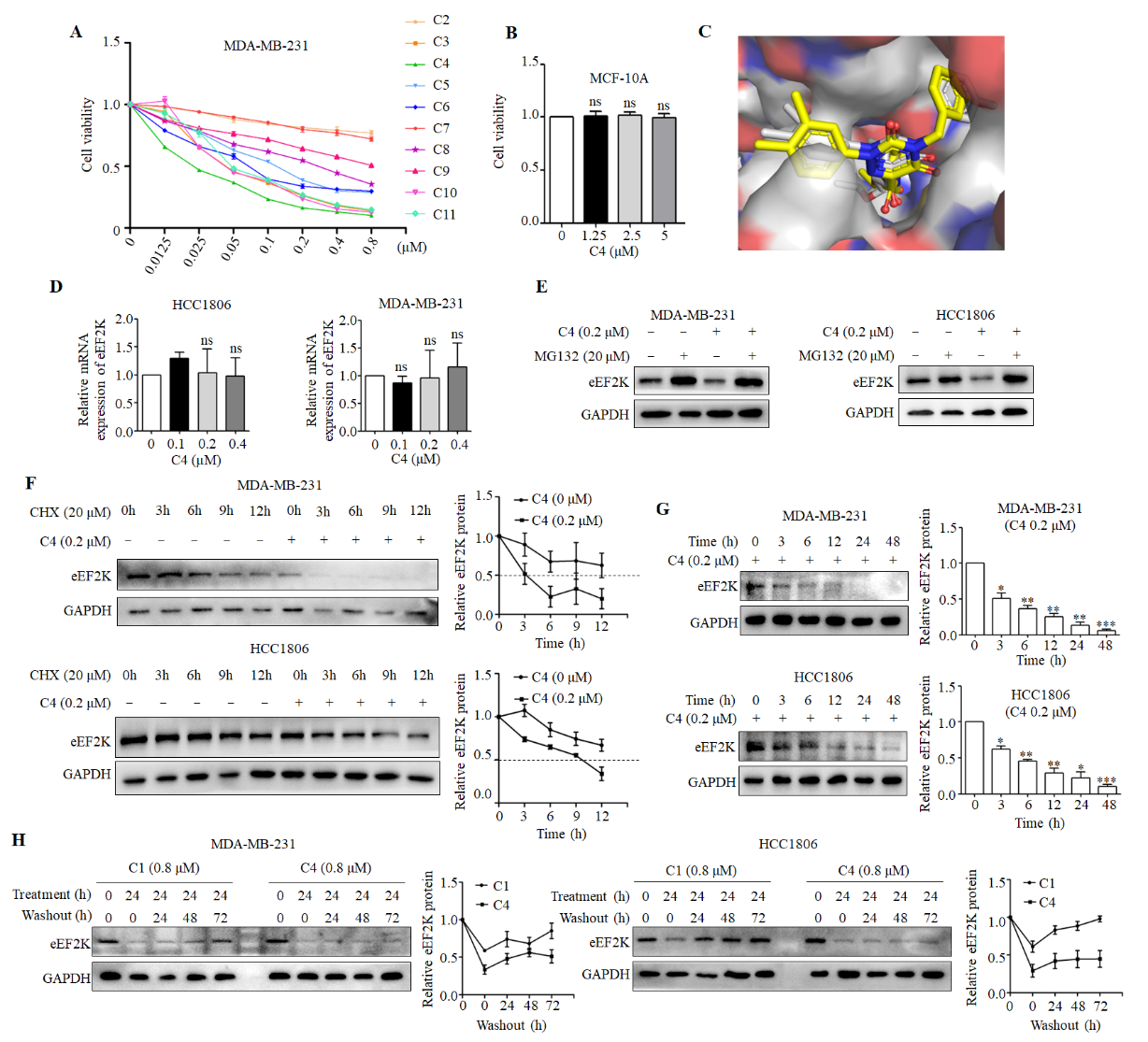


**Figure S7. Compound 4 promotes the degradation of eEF2K in TNBC cells**

**(A)** Cell viability of MDA-MB-231 cells treated with C1 analogs at different concentrations. **(B)** Cell viability of MCF-10A cells treated with C4 at different concentrations. ns, no significance. **(C)** The binding modes of compounds C4 (yellow) and C1 (white) to the homology model of eEF2K. **(D)** Expression of eEF2K mRNA in MDA-MB-231 and HCC1806 cells treated with C4. ns, no significance. **(E)** 20 μM MG132 was added to MDA-MB-231 and HCC1806 cells treated with C4 for 24 h. The expressions of eEF2K were detected by western blot assay. **(F)** MDA-MB-231 and HCC1806 cells pretreated with or without C4 were treated with cycloheximide (CHX) (10 μg/ml) for the indicated time. The expressions of eEF2K western blot were detected by western blot assay. **(G)** eEF2K protein expression was detected in MDA-MB-231 and HCC1806 cells treated with 0.2 μM C4 for different time. ****P* < 0.001, *****P* < 0.01, **P* < 0.05. **(H)** MDA-MB-231 and HCC1806 cells were treated with 0.8 μM C4 or C1 for 24 h, and then washed away the compounds with PBS and received the protein at the indicated time. eEF2K was detected by western blot.


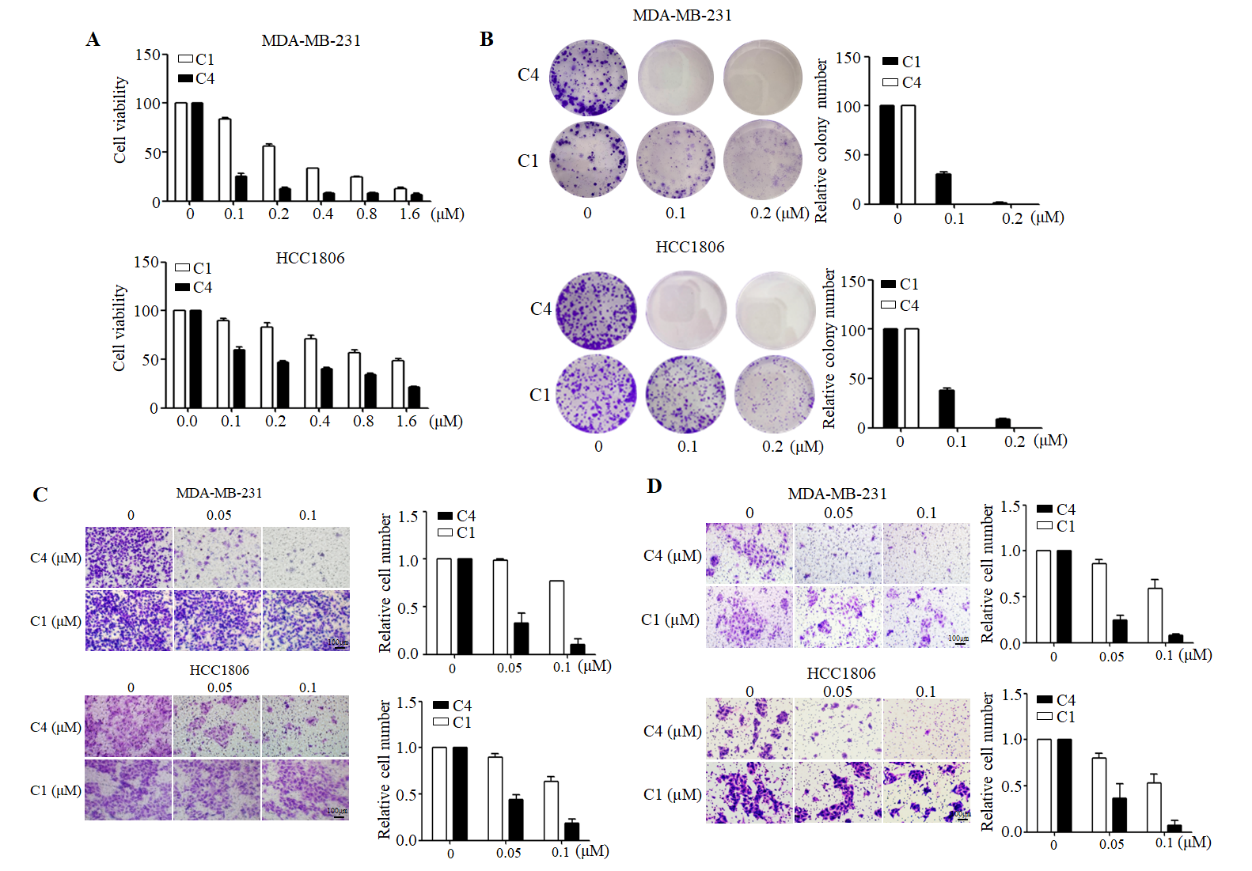


**Figure S8. Comparison of the extracellular antitumor activity of C1 and C4**

**(A)** Cell viability of MDA-MB-231 and HCC1806 cells treated with C4 or C1 at different concentrations. **(B)** Numbers of colonies of MDA-MB-231 and HCC1806 cells were detected by colony formation assay after treated with C4 or C1. **(C-D)** The transwell assay compared the effects of C1 and C4 on cell migration (C) and invasion (D). Scale bar, 100 μm.


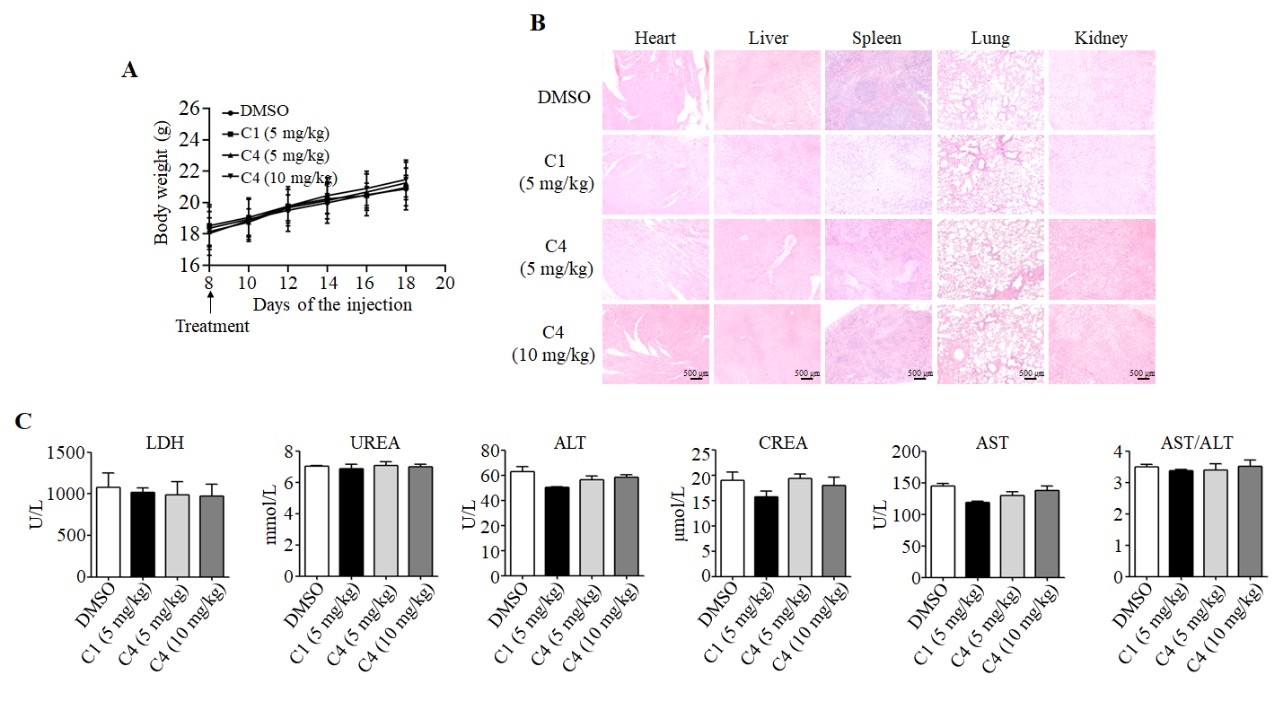


**Figure S9.** **Intravenous toxicity assessment of compounds C1 and C4**

**(A)** The effect of C1 or C4 treatment on mice body weight. **(B)** H&E staining was used to detect the morphology of heart, liver, spleen, lung and kidney of mice. Scale bar, 500 μm. **(C)** The liver and kidney function of mice were measured by the serum of mice.

**Table S1. Primers of qRT-PCR.**

| Primer | Sequence (5’-3’) |
| --- | --- |
| eEF2K-Forward | TGCGAGAAGGGCGAGGAGTG |
| eEF2K-Reverse | GGCTAGGATGTGATGAGGCAACTG |
| SOX8-Forward | GGTGCTCAAGGGCTACGACTG |
| SOX8-Reverse | TTCACATGCGGCTTGGCTTTG |
| GAPDH-Forward | TGACATCAAGAAGGTGGTGAAGCAG |
| GAPDH-Reverse | GTGTCGCTGTTGAAGTCAGAGGAG |

**Table S2. Target sequences of siRNA and shRNA.**

| siRNA/shRNA | Sequence (5’-3’) |
| --- | --- |
| Human sieEF2K 1# | GCUCGAACCAGAAUGUCAA |
| Human sieEF2K 2# | GCAAACUCCUUCCACUUCA |
| Human sheEF2K 1# | CCTGGAAGTGCAAAGGCTT |
| Human sheEF2K 2# | GCTCGAACCAGAATGTCAA |
| Human shAURKA 1# | GAGTCTACCTAATTCTGGAAT |
| Human shAURKA 2# | CCTGTCTTACTGTCATTCGAA |

**Table S3. The correlation between eEF2K expression and clinicopathological characteristics in tissue microarray of 80 TNBC patients.**

| **Characteristic** | **Total** | | **eEF2K expression** | | | **χ^2^** | | ***P*** |
| --- | --- | --- | --- | --- | --- | --- | --- | --- |
|  |  |  | **Low**  **(n=43)** | **High**  **(n=37)** | |  |  |  |
| **Lymph node metastasis** | | | | | 16.441 | | **＜0.0001** | |
| Yes | | 41 | 13 | 28 | |  | |  |
| No | | 39 | 30 | 9 | |  | |  |
| **Distant metastasis** | | | | | 6.084 | | **0.0136** | |
| M0 | | 72 | 42 | 30 | |  | |  |
| M1 | | 8 | 1 | 7 | |  | |  |
| **TNM stage** | | | | | 9.442 | | **0.0021** | |
| Ⅰ-Ⅱ | | 51 | 34 | 17 | |  | |  |
| Ⅲ-Ⅳ | | 29 | 9 | 20 | |  | |  |
| **Age (years)** | | | | | 0.0503 | | 0.8226 | |
| < 50 | | 40 | 22 | 18 | |  | |  |
| ≥ 50 | | 40 | 21 | 19 | |  | |  |
| **Ki67 (%)** | | | | | 2.905 | | 0.1478 | |
| < 50 | | 30 | 13 | 17 | |  | |  |
| ≥ 50 | | 50 | 30 | 20 | |  | |  |
| **Tumor size (Maximum diameter)** | | | | | 9.483 | | **0.0021** | |
| ≤ 3cm | | 45 | 31 | 14 | |  | |  |
| > 3cm | | 35 | 12 | 23 | |  | |  |

**Table S4. The correlation between SOX8 expression and clinicopathological characteristics in tissue microarray of 80 TNBC patients.**

| **Characteristic** | **Total** | | **SOX8 expression** | | | **χ^2^** | | ***P*** |
| --- | --- | --- | --- | --- | --- | --- | --- | --- |
|  |  |  | **Low**  **(n=48)** | **High**  **(n=32)** | |  |  |  |
| **Lymph node metastasis** | | | | | 9.081 | | **0.0026** | |
| Yes | | 41 | 18 | 23 | |  | |  |
| No | | 39 | 30 | 9 | |  | |  |
| **Distant metastasis** | | | | | 8.039 | | **0.0046** | |
| M0 | | 72 | 47 | 26 | |  | |  |
| M1 | | 8 | 1 | 7 | |  | |  |
| **TNM stage** | | | | | 4.363 | | **0.0367** | |
| Ⅰ-Ⅱ | | 51 | 35 | 16 | |  | |  |
| Ⅲ-Ⅳ | | 29 | 13 | 16 | |  | |  |
| **Age (years)** | | | | | 0.208 | | 0.6481 | |
| < 50 | | 40 | 25 | 15 | |  | |  |
| ≥ 50 | | 40 | 23 | 17 | |  | |  |
| **Ki67 (%)** | | | | | 0.889 | | 0.3458 | |
| < 50 | | 30 | 16 | 14 | |  | |  |
| ≥ 50 | | 50 | 32 | 18 | |  | |  |
| **Tumor size (Maximum diameter)** | | | | | 3.386 | | 0.0657 | |
| ≤ 3cm | | 45 | 31 | 14 | |  | |  |
| > 3cm | | 35 | 17 | 18 | |  | |  |

**Table S5. The correlation between p-AURKA (Ser391) expression and clinicopathological characteristics in tissue microarray of 80 TNBC patients.**

| **Characteristic** | **Total** | | **p-AURKA (Ser391) expression** | | | **χ^2^** | | ***P*** |
| --- | --- | --- | --- | --- | --- | --- | --- | --- |
|  |  |  | **Low**  **(n=44)** | **High**  **(n=36)** | |  |  |  |
| **Lymph node metastasis** | | | | | 4.185 | | **0.0408** | |
| Yes | | 41 | 18 | 23 | |  | |  |
| No | | 39 | 26 | 13 | |  | |  |
| **Distant metastasis** | | | | | 6.487 | | **0.0109** | |
| M0 | | 72 | 43 | 29 | |  | |  |
| M1 | | 8 | 1 | 7 | |  | |  |
| **TNM stage** | | | | | 5.355 | | **0.0207** | |
| Ⅰ-Ⅱ | | 51 | 33 | 18 | |  | |  |
| Ⅲ-Ⅳ | | 29 | 11 | 18 | |  | |  |
| **Age (years)** | | | | | 0.808 | | 0.3687 | |
| < 50 | | 40 | 20 | 20 | |  | |  |
| ≥ 50 | | 40 | 24 | 16 | |  | |  |
| **Ki67 (%)** | | | | | 0.054 | | 0.8165 | |
| < 50 | | 30 | 16 | 14 | |  | |  |
| ≥ 50 | | 50 | 28 | 22 | |  | |  |
| **Tumor size (Maximum diameter)** | | | | | 0.321 | | 0.5712 | |
| ≤ 3cm | | 45 | 26 | 19 | |  | |  |
| > 3cm | | 35 | 18 | 17 | |  | |  |

**Table S6. Inhibitory effects of C1 analogs on cell viability in MDA-MB-231 cells.**

| **cpd** | **R_4_** | **MDA-MB-231**  **Inhibition (%)**  **@ 20 μM** | **MDA-MB-231**  **IC_50_ (nM)** | **cLog P** | **Log S** | **tPSA** | **SPR Avg KD (nM)** |
| --- | --- | --- | --- | --- | --- | --- | --- |
| **C1** | 3-F-Bn | 98.44% | 131.2 | 3.77 | -6.26 | 99.59 | 1.26 |
| **C2** | CH_3_ | 69.16% | > 10000 | 1.86 | -4.45 | 99.59 | - |
| **C3** | 3-Cl-Bn | 89.54% | 59.2 | 4.34 | -6.68 | 99.59 | 19.9 |
| **C4** | 3-Me-Bn | 98.90% | 24.46 | 4.13 | -6.34 | 99.59 | 831 |
| **C5** | 3-CF_3_-Bn | 95.19% | 128.9 | 4.51 | -7.00 | 99.59 | 16500 |
| **C6** | 3-OMe-Bn | 92.97% | 82.77 | 3.55 | -6.04 | 108.82 | 25.4 |
| **C7** | 3-CH_3_SO_2_-Bn | 89.77% | > 1000 | -0.27 | -5.757 | 133.65 | - |
| **C8** | 3-*i*-Pr-Bn | 89.96% | 60.11 | 5.05 | -7.082 | 99.59 | 171 |
| **C9** | 2-Me-Bn | 92.02% | 259.2 | 4.08 | -6.35 | 99.59 | 546 |
| **C10** | 4-Me-Bn | 91.52% | 544.3 | 4.13 | -6.34 | 99.59 | 8.82E-04 |
| **C11** | 3,5-di-Me-Bn | 90.13% | 69.61 | 4.63 | -6.70 | 99.59 | 1.31E-06 |

**Supplementary Information for**

**Identification of a novel substrate for eEF2K and the AURKA-SOX8 as the related pathway in TNBC**

Xiaoya Wan et al.

Corresponding author: Yan Cheng, yancheng@csu.edu.cn

**Table of Content**

[**Scheme S1 2**](#_Toc2597)

[**Synthetic procedure for 2-(3,4-dimethylphenyl)-4-methyl-3,5-dioxo-2,3,4,5-tetrahydro-1,2,4-triazine-6-carbonitrile 2a 3**](#_Toc27366)

[**General procedure for the synthesis of compounds 2b-2j 3**](#_Toc2123)

[**General procedure for the synthesis of compounds C2-C11 5**](#_Toc2904)

[**^1^H and ^13^C NMR data 12**](#_Toc6639)

**Scheme S1**. The synthesis of C1 analogs. Reagents and conditions: (a) K_2_CO_3_, substituted benzyl bromide or benzyl bromide, acetonitrile, r.t, overnight, 52-79%. (b) i) Concentrated HCl, AcOH, 110°C, 68-80%; ii) DCM, oxalyl chloride, N_2_, 0℃, overnight; iii) Ethyl piperidine-3-carboxylate, DCM, r.t, overnight, 40-69%.

**General Information**

All commercially available starting materials and solvents were of reagent grade and used without further purification. Reactions were performed under a nitrogen atmosphere in dry glassware with magnetic stirring. Column chromatography was carried out on silica gel (200–300 mesh). All reactions were monitored by UV (254 nm) via thin-layer chromatography (TLC) on silica gel plates. NMR spectra were recorded on a Bruker-600, Bruker-400 or **Vnmrs**-300 (^1^H, 400 and 300 MHz; ^13^C, 151 and 101 MHz) spectrometer. ^1^H and ^13^C NMR spectra were recorded with tetramethylsilane (TMS) as an internal reference. Chemical shifts are expressed in parts per million, and *J* values are given in Hz. High-resolution mass spectra (HRMS) were obtained using a Waters Xevo G2-XS Tof (ESI) mass spectrometer and a GCT Premier TM (EI) mass spectrometer. The purities of the final compounds were established by analytical HPLC. HPLC analysis conditions: COSMOSIL 5C18-MS-II, 3.5 μm, 4.6 mm × 250 mm; flow rate 1 mL/min; UV detection at 254 nm; linear gradient from 10% ACN in water to 100% ACN in water in 30 min. All biologically evaluated compounds were >95% pure.

**Synthetic procedure for 2-(3,4-dimethylphenyl)-4-methyl-3,5-dioxo-2,3,4,5-tetrahydro-1,2,4-triazine-6-carbonitrile (2a).**

To a solution of compound **1** (400 mg, 1.65 mmol) and potassium carbonate (456 mg, 3.30mmol) in acetonitrile, iodomethane was added dropwise and stirred for 4 h. Upon completion of the reaction as indicated by TLC, the reaction mixture was decompressed, evaporated, and extracted with water (8 mL) and DCM (12 mL). The organic phase was concentrated, dried over anhydrous NaSO_4_, and then filtrated. The filtrates were evaporated and purified using silica gel chromatography to obtain the title compound **2a** as a white solid in 52% yield (220 mg). ^1^H NMR (300 MHz, CDCl_3_) δ 7.34 – 7.13 (m, 3H), 3.46 (s, 3H), 2.32 (s, 6H).

**General procedure for the synthesis of compounds 2b-2j**

To a solution of compound **1** (0.41 mmol) and potassium carbonate (0.83mmol) in acetonitrile, various (bromomethyl)benzene (0.50 mmol) were added dropwise and stirred for 4 h. Upon completion of the reaction as indicated by TLC, the reaction mixture was decompressed, evaporated, and extracted with water (2 mL) and DCM (3 mL). The organic phase was concentrated, recrystallized, filtered, and then dried to give the title compounds **2b-2j.**

**4-(3-Chlorobenzyl)-2-(3,4-dimethylphenyl)-3,5-dioxo-2,3,4,5-tetrahydro-1,2,4-triazine-6-carbonitrile (2b).** Compound **2b** was prepared as a white solid (114 mg) in 75% yield from **1** (100 mg, 0.41 mmol) and 1-(bromomethyl)-3-chlorobenzene (102 mg, 0.50 mmol). ^1^H NMR (300 MHz, CDCl_3_) δ 7.52 (s, 1H), 7.42 (d, *J* = 7.0 Hz, 1H), 7.35 – 7.14 (m, 5H), 5.12 (s, 2H), 2.31 (s, 6H).

**2-(3,4-Dimethylphenyl)-4-(3-methylbenzyl)-3,5-dioxo-2,3,4,5-tetrahydro-1,2,4-triazine-6-carbonitrile (2c)**. Compound **2c** was prepared as a white solid (102 mg) in 71% yield from **1** (87 mg, 0.36 mmol) and 1-(bromomethyl)-3-methylbenzene (80 mg, 0.43 mmol). ^11^H NMR (300 MHz, CDCl_3_) δ 7.41 (d, *J* = 5.8 Hz, 2H), 7.34-7.21 (m, 5H), 5.20 (s, 2H), 2.41 (s, 3H), 2.39 (s, 6H).

**2-(3,4-Dimethylphenyl)-3,5-dioxo-4-(3-(trifluoromethyl)benzyl)-2,3,4,5-tetrahydro-1,2,4-triazine-6-carbonitrile (2d).** Compound **2d** was prepared as a white solid (112 mg) in 68% yield from **1** (100 mg, 0.41 mmol) and 1-(bromomethyl)-3-(trifluoromethyl)benzene (118 mg, 0.50 mmol). ^1^H NMR (400 MHz, CDCl_3_) δ 7.79 (s, 1H), 7.74 (d, *J* = 7.7 Hz, 1H), 7.61 (d, *J* = 7.8 Hz, 1H), 7.48 (t, *J* = 7.8 Hz, 1H), 7.26 (s, 2H), 7.16 (d, *J* = 2.4 Hz, 1H), 5.21 (s, 2H), 2.31 (s, 6H).

**2-(3,4-Dimethylphenyl)-4-(3-methoxybenzyl)-3,5-dioxo-2,3,4,5-tetrahydro-1,2,4-triazine-6-carbonitrile (2e).** Compound **2e** was prepared as a white solid (350 mg) in 68% yield from **1** (345 mg, 1.42 mmol) and 1-(bromomethyl)-3-methoxybenzene (344 mg, 1.71 mmol). ^1^H NMR (300 MHz, CDCl_3_) δ 7.32 (dd, *J* = 15.5, 7.9 Hz, 4H), 7.20 (d, *J* = 14.2 Hz, 2H), 7.04 – 6.88 (m, 1H), 5.23 (s, 2H), 3.90 (s, 3H), 2.41 (s, 6H).

**2-(3,4-Dimethylphenyl)-4-(3-(methylsulfonyl)benzyl)-3,5-dioxo-2,3,4,5-tetrahydro-1,2,4-triazine-6-carbonitrile (2f).** Compound **2f** was prepared as a white solid (134 mg) in 79% yield from **1** (100 mg, 0.41 mmol) and 1-(bromomethyl)-3-(methylsulfonyl)benzene (123 mg, 0.50 mmol). ^1^H NMR (300 MHz, CDCl_3_) δ 8.07 (s, 1H), 7.91 (d, *J* = 8.1 Hz, 1H), 7.82 (d, *J* = 7.8 Hz, 1H), 7.56 (t, *J* = 7.9 Hz, 1H), 7.22 – 7.14 (m, 3H), 5.22 (s, 2H), 3.05 (s, 3H), 2.31 (s, 6H).

**2-(3,4-Dimethylphenyl)-4-(3-isopropylbenzyl)-3,5-dioxo-2,3,4,5-tetrahydro-1,2,4-triazine-6-carbonitrile (2g).** Compound **2g** was prepared as a white solid (107 mg) in 69% yield from **1** (100 mg, 0.41 mmol) and 1-(bromomethyl)-3-(isopropyl benzyl)benzene (93 mg, 0.50 mmol). ^1^H NMR (300 MHz, CDCl_3_) δ 7.49 – 7.03 (m, 7H), 5.11 (s, 2H), 2.85 (h, *J* = 7.1 Hz, 1H), 2.27 (s, 6H), 1.20 (d, *J* = 7.0 Hz, 6H).

**2-(3,4-Dimethylphenyl)-4-(2-methylbenzyl)-3,5-dioxo-2,3,4,5-tetrahydro-1,2,4-triazine-6-carbonitrile (2h).** Compound **2h** was prepared as a white solid (90 mg) in 63% yield from **1** (100 mg, 0.41 mmol) and 1-(bromomethyl)-2-(methylbenzyl)benzene (92 mg, 0.50 mmol). ^1^H NMR (300 MHz, CDCl_3_) δ 7.35 – 7.01 (m, 7H), 5.19 (s, 2H), 2.48 (s, 3H), 2.30 (s, 6H).

**2-(3,4-Dimethylphenyl)-4-(4-methylbenzyl)-3,5-dioxo-2,3,4,5-tetrahydro-1,2,4-triazine-6-carbonitrile (2i).**Compound **2i** was prepared as a white solid (82 mg) in 57% yield from **1** (100 mg, 0.41 mmol) and 1-(bromomethyl)-4-(methylbenzyl)benzene (92 mg, 0.50 mmol). ^1^H NMR (300 MHz, CDCl_3_) δ 7.47 (d, *J* = 7.8 Hz, 2H), 7.32 – 7.13 (m, 5H), 5.16 (s, 2H), 2.38 (s, 3H), 2.35 (s, 6H).

**4-(3,5-Dimethylbenzyl)-2-(3,4-dimethylphenyl)-3,5-dioxo-2,3,4,5-tetrahydro-1,2,4-triazine-6-carbonitrile (2j).** Compound **2j** was prepared as a white solid (94 mg) in 63% yield from **1** (100 mg, 0.41 mmol) and 1-(bromomethyl)-3,5-dimethylbenzene (99 mg, 0.50 mmol).^1^H NMR (300 MHz, CDCl_3_) δ 7.39 – 7.24 (m, 5H), 7.09 (s, 1H), 5.20 (s, 2H), 2.43 (s, 6H), 2.41 (s, 6H).

**General procedure for the synthesis of compounds C2-C11.**

Compounds **2a-2j** were dissolved in a mixture of concentrated hydrochloric acid (1 mL) and glacial acetic acid (2.5 mL) and refluxed for 4 h. The solvent was evaporated in vacuo, and then water (3 mL) was added. Subsequently, the precipitate was filtrated and stirred in anhydrous ether (2mL) for 10 min. Corresponding acids were obtained by filtration and dryness under vacuum without purification as a white solid.

To a solution of the Corresponding acid in DCM (2 mL), one drop of DMF and oxalyl dichloride (0.03 mL) was added dropwise under the atmosphere of N_2_ and then stirred at room temperature for 3 h. The reaction mixture was concentrated to dryness to obtain the corresponding acyl chlorides as yellow solids.

The above acyl chloride was added dropwise to a solution of ethyl piperidine-3-carboxylate (1.2 eq) and Et_3_N (2 eq) in DCM (2 mL), while maintaining the temperature below 0 °C. Then, the reaction mixture was stirred at rt for 3 h. Upon completion of the reaction as indicated by TLC, the mixture was washed successively with water (5 mL × 3) and brine (5 mL × 2), dried over anhydrous NaSO_4_, and then filtrated. The filtrates were evaporated and purified using silica gel chromatography to obtain the title compounds **C2-C11**.

**Ethyl 1-(2-(3,4-dimethylphenyl)-4-methyl-3,5-dioxo-2,3,4,5-tetrahydro-1,2,4-triazine-6-carbonyl)piperidine-3-carboxylate (C2).** Compound **C2** was prepared as a white solid (170 mg) in 37% yield from **2a** (284 mg, 1.11 mmol) and ethyl piperidine-3-carboxylate (137 mg, 0.87 mmol). ^1^H NMR (300 MHz, CDCl_3_) δ 7.25 – 7.13 (m, 3H), 4.69 (d, *J* = 12.8 Hz, 0.5H), 4.41 (d, *J* = 13.3 Hz, 0.5H), 4.18 – 4.05 (m, 2H), 3.77 (d, *J* = 12.9 Hz, 0.5H), 3.58 (d, *J* = 13.9 Hz, 0.5H), 3.41 (s, 3H), 3.35 – 3.25 (m, 0.5H), 3.20 – 3.03 (m, 1H), 3.01 – 2.87 (m, 0.5H), 2.64 – 2.55 (m, 1H), 2.27 (s, 6H), 2.18 – 2.09 (m, 1H), 1.78 – 1.56 (m, 3H), 1.27 – 1.19 (m, 3H). ^13^C NMR (101 MHz, CDCl_3_) δ 172.8, 172.7, 160.3, 154.1, 154.0, 148.3, 148.2, 139.2, 138.9, 137.6, 137.6, 137.5, 130.1, 130.1, 126.3, 126.2, 122.6, 122.5, 61.0, 60.9, 48.7, 47.4, 43.8, 42.5, 41.7, 41.1, 27.7, 27.4, 27.3, 24.9, 24.0, 19.9, 19.9, 19.5, 14.2, 14.2.HRMS (ESI) calcd for C_21_H_27_N_4_O_5_ [M+H]^+^: 415.1981, found 415.1982. HPLC purity 97.59% (t_R_ = 9.78 min).

**Ethyl 1-(4-(3-chlorobenzyl)-2-(3,4-dimethylphenyl)-3,5-dioxo-2,3,4,5-tetrahydro-1,2,4-triazine-6-carbonyl)piperidine-3-carboxylate** **(C3).** Compound **C3** was prepared as a white solid (57 mg) in 35% yield from **2b** (114 mg, 0.32 mmol) and ethyl piperidine-3-carboxylate (26 mg, 0.17 mmol). ^1^H NMR (300 MHz, CDCl_3_) δ 8.15 (d, *J* = 8.2 Hz, 0.35H), 7.94 (d, *J* = 8.3 Hz, 0.3H), 7.64 (d, *J* = 8.2 Hz, 1H), 7.49 (d, *J* = 7.1 Hz, 1H), 7.39 – 7.25 (m, 4H), 6.43 (s, 0.35H), 5.18 (s, 2H), 4.77 (d, *J* = 13.3 Hz, 0.5H), 4.43 (d, *J* = 13.4 Hz, 0.5H), 4.23 (q, *J* = 5.2, 4.1 Hz, 2H), 3.80 (d, *J* = 13.5 Hz, 0.5H), 3.62 (d, *J* = 13.5 Hz, 0.5H), 3.43 (dd, *J* = 13.6, 10.0 Hz, 0.5H), 3.29 – 2.99 (m, 1.5H), 2.72 – 2.62 (m, 1H), 2.35 (s, 6H), 2.21 (d, *J* = 11.3 Hz, 1H), 1.95 – 1.69 (m, 3H), 1.37 – 1.23 (m, 3H). ^13^C NMR (151 MHz, CDCl_3_) δ 172.8, 172.7, 160.2, 153.8, 153.8, 148.1, 148.0, 143.6, 139.6, 139.3, 137.7, 137.4, 137.4, 136.8, 134.6, 134.5, 130.1, 130.0, 129.1, 128.78, 128.7, 128.3, 128.3, 126.3, 126.2, 125.4, 122.7, 122.6, 120.4, 109.4, 105.01, 61.1, 61.0, 48.8, 47.5, 44.3, 44.2, 43.9, 42.5, 41.7, 41.2, 27.4, 27.4, 24.9, 24.0, 20.0, 20.0, 14.3, 14.3. HRMS (ESI) calcd for C_27_H_29_N_4_O_5_NaCl [M+Na]^+^: 547.1724, found 547.1729. HPLC purity 98.64% (t_R_ = 12.20 min).

**Ethyl 1-(2-(3,4-dimethylphenyl)-4-(3-methylbenzyl)-3,5-dioxo-2,3,4,5-tetrahydro-1,2,4-triazine-6-carbonyl)piperidine-3-carboxylate** (**C4**). Compound **C4** was prepared as a white solid (83 mg) in 52% yield from **2c** (110 mg, 0.32 mmol) and ethyl piperidine-3-carboxylate (43 mg, 0.27 mmol). ^1^H NMR (300 MHz, CDCl_3_) δ 7.32 (d, *J* = 6.5 Hz, 2H), 7.28 – 7.22 (m, 1H), 7.19 (d, *J* = 4.5 Hz, 3H), 7.10 (d, *J* = 7.5 Hz, 1H), 5.11 (s, 2H), 4.71 (d, *J* = 13.3 Hz, 0.5H), 4.40 (d, *J* = 13.2 Hz, 0.5H), 4.24 – 3.99 (m, 2H), 3.75 (d, *J* = 13.8 Hz, 0.5H), 3.55 (d, *J* = 13.8 Hz, 0.5H), 3.41 – 3.23 (m, 0.5H), 3.17 – 2.87 (m, 1.5H), 2.60 (qt, *J* = 8.0, 3.4 Hz, 1H), 2.31 (s, 3H), 2.27 (s, 6H), 2.14 (d, *J* = 7.9 Hz, 1H), 1.89 – 1.53 (m, 3H), 1.42 – 1.07 (m, 3H). ^13^C NMR (101 MHz, CDCl_3_) δ 172.9, 172.7, 160.4, 154.0, 153.9, 148.2, 148.1, 139.7, 139.4, 138.5, 138.5, 137.7, 137.7, 137.6, 137.6, 137.5, 135.0, 135.0, 130.8, 130.7, 130.1, 130.1, 129.3, 129.2, 128.7, 128.6, 127.2, 127.1, 126.4, 126.3, 122.8, 122.7, 61.0, 61.0, 48.8, 47.5, 44.9, 44.8, 43.9, 42.5, 41.8, 41.2, 27.5, 27.4, 24.9, 24.1, 21.5, 20.0, 20.0, 19.6, 14.3, 14.3. HRMS (ESI) calcd for C_28_H_32_N_4_O_5_ [M]^+^: 504.2373, found 504.2371. HPLC purity 98.48% (t_R_ = 12.14 min).

**Ethyl 1-(2-(3,4-dimethylphenyl)-3,5-dioxo-4-(3-(trifluoromethyl)benzyl)-2,3,4,5-tetrahydro-1,2,4-triazine-6-carbonyl)piperidine-3-carboxylate** (**C5**). Compound **C5** was prepared as a white solid (150 mg) in 35% yield from **2d** (305 mg, 0.76 mmol) and ethyl piperidine-3-carboxylate (75 mg, 0.48 mmol). ^1^H NMR (300 MHz, CDCl_3_) δ 7.80 (s, 1H), 7.74 (d, *J* = 7.7 Hz, 1H), 7.57 (d, *J* = 7.8 Hz, 1H), 7.45 (t, *J* = 7.7 Hz, 1H), 7.23 (d, *J* = 5.3 Hz, 1H), 7.20 (s, 2H), 5.20 (s, 2H), 4.78 – 4.60 (m, 0.5H), 4.46 – 4.28 (m, 0.5H), 4.25 – 4.00 (m, 2H), 3.74 (dd, *J* = 13.8, 4.0 Hz, 0.5H), 3.54 (d, *J* = 13.2 Hz, 0.5H), 3.37 (dd, *J* = 13.7, 9.8 Hz, 0.5H), 3.22 – 2.94 (m, 1.5H), 2.68 – 2.53 (m, 1H), 2.29 (s, 6H), 2.15 (d, *J* = 10.9 Hz, 1H), 1.93 – 1.63 (m, 3H), 1.24 (dt, *J* = 14.4, 7.1 Hz, 3H). ^13^C NMR (151 MHz, CDCl_3_) δ 172.7 (d, *J_C-F_* = 16.9 Hz), 160.2 (d, *J_C-F_* = 3.2 Hz), 153.8 (d, *J_C-F_* = 5.7 Hz), 148.1 (d, *J_C-F_* = 9.7 Hz), 139.6, 139.3, 137.8, 137.4 (d, *J_C-F_* = 7.5 Hz), 133.6 (d, *J_C-F_* = 9.0 Hz), 131.2, 131.0 (d, *J_C-F_* = 8.4 Hz), 130.1, 129.3 (d, *J_C-F_* = 3.6 Hz), 126.9 (d, *J_C-F_* = 4.0 Hz), 126.8 (d, *J_C-F_* = 4.0 Hz), 126.3, 126.2, 125.4, 122.7, 122.6, 61.0, 61.0, 48.8, 47.5, 44.4, 44.3, 43.9, 42.5, 41.7, 41.2, 27.4, 27.4, 24.8, 24.0, 20.0, 19.9, 19.6, 14.3, 14.2. HRMS (ESI) calcd for C_28_H_29_N_4_O_5_F_3_ [M]^+^: 558.2090, found 558.2085. HPLC purity 97.52% (t_R_ = 12.25 min).

**Ethyl 1-(2-(3,4-dimethylphenyl)-4-(3-methoxybenzyl)-3,5-dioxo-2,3,4,5-tetrahydro-1,2,4-triazine-6-carbonyl)piperidine-3-carboxylateb** (**C6**). Compound **C6** was prepared as a white solid (311 mg) in 52% yield from **2e** (420 mg, 1.16 mmol) and ethyl piperidine-3-carboxylate (118 mg, 0.82 mmol). ^1^H NMR (400 MHz, CDCl_3_) δ 7.33 – 7.15 (m, 4H), 7.13 – 7.03 (m, 2H), 6.92 – 6.79 (m, 1H), 5.13 (s, 2H), 4.81 – 4.65 (m, 0.5H), 4.40 (d, *J* = 13.3 Hz, 0.5H), 4.25 – 4.02 (m, 2H), 3.78 (s, 3H), 3.75 – 3.67 (m, 0.5H), 3.62 – 3.50 (m, 0.5H), 3.34 (dd, *J* = 13.7, 10.1 Hz, 0.5H), 3.18 – 2.93 (m, 1.5H), 2.74 – 2.52 (m, 1H), 2.28 (s, 6H), 2.19 – 2.08 (m, 1H), 1.89 – 1.55 (m, 3H), 1.24 (dt, *J* = 18.4, 7.1 Hz, 3H). ^13^C NMR (101 MHz, CDCl_3_) δ 172.8, 172.7, 160.3, 159.8, 159.8, 153.9, 153.8, 148.1, 148.1, 139.6, 139.3, 137.6, 137.6, 137.5, 137.5, 136.4, 136.4, 130.0, 130.0, 129.7, 129.7, 126.3, 126.2, 122.7, 122.6, 122.2, 122.2, 115.3, 114.2, 61.0, 61.0, 55.3, 55.3, 48.7, 47.4, 44.8, 44.7, 43.8, 42.5, 41.7, 41.1, 27.4, 27.4, 24.9, 24.0, 19.9, 19.9, 19.5, 14.2, 14.2. HRMS (EI) calcd for C_28_H_32_N_4_O_6_ [M]^+^: 520.2322, found 520.2326. HPLC purity 97.63% (t_R_ = 11.69 min).

**Ethyl 1-(2-(3,4-dimethylphenyl)-4-(3-(methylsulfonyl)benzyl)-3,5-dioxo-2,3,4,5-tetrahydro-1,2,4-triazine-6-carbonyl)piperidine-3-carboxylate** **(C7)**. Compound **C7** was prepared as a white solid (106 mg) in 27% yield from **2f** (134 mg, 0.34 mmol) and ethyl piperidine-3-carboxylate (59 mg, 0.38 mmol). ^1^H NMR (300 MHz, CDCl_3_) δ 8.07 (d, *J* = 7.1 Hz, 1H), 7.92 – 7.73 (m, 2H), 7.52 (t, *J* = 7.8 Hz, 1H), 7.22 (d, *J* = 4.8 Hz, 1H), 7.18 (s, 2H), 5.21 (s, 2H), 4.74 – 4.61 (m, 0.5H), 4.32 (d, *J* = 13.2 Hz, 0.5H), 4.22 – 4.01 (m, 2H), 3.81 – 3.69 (m, 0.5H), 3.57 (d, *J* = 13.7 Hz, 0.5H), 3.37 (dd, *J* = 13.7, 9.8 Hz, 0.5H), 3.23 – 2.93 (m, 4.5H), 2.60 (s, 1H), 2.27 (s, 6H), 2.13 (d, *J* = 12.1 Hz, 1H), 1.78 – 1.53 (m, 3H), 1.23 (dt, *J* = 12.6, 7.1 Hz, 3H). ^13^C NMR (151 MHz, CDCl_3_) δ 172.8, 172.7, 160.1, 153.8, 148.1, 148.0, 141.2, 141.2, 139.6, 139.2, 137.8, 137.4, 137.3, 136.7, 136.6, 135.3, 135.3, 130.1, 129.9, 128.9, 128.7, 127.4, 126.3, 126.2, 122.6, 122.6, 61.1, 61.0, 48.8, 47.6, 44.5, 44.5, 44.2, 44.2, 43.9, 42.6, 41.7, 41.2, 27.4, 24.9, 24.0, 20.0, 20.0, 19.6, 14.3, 14.2. HRMS (ESI) calcd for C_28_H_32_N_4_O_7_NaS [M+Na]^+^: 591.1889, found 591.1893. HPLC purity 97.44% (t_R_ = 10.65 min).

**Ethyl 1-(2-(3,4-dimethylphenyl)-4-(3-isopropylbenzyl)-3,5-dioxo-2,3,4,5-tetrahydro-1,2,4-triazine-6-carbonyl)piperidine-3-carboxylate** (**C8**). Compound **C8** was prepared as a white solid (85 mg) in 55% yield from **2g** (107 mg, 0.30 mmol) and ethyl piperidine-3-carboxylate (54 mg, 0.34 mmol). ^1^H NMR (300 MHz, CDCl_3_) δ 7.58 – 7.36 (m, 2H), 7.35 – 6.94 (m, 5H), 5.21 (s, 2H), 4.89 – 4.62 (m, 0.5H), 4.48 (d, *J* = 13.3 Hz, 0.5H), 4.32 – 4.05 (m, 2H), 3.95 – 3.73 (m, 0.5H), 3.68 – 3.49 (m, 1H), 3.45 – 3.32 (m, 0.5H), 3.26 – 2.86 (m, 2H), 2.78 – 2.58 (m, 1H), 2.35 (s, 6H), 2.27 – 2.12 (m, 1H), 1.96 – 1.61 (m, 3H), 1.43 – 1.14 (m, 9H). ^13^C NMR (151 MHz, CDCl_3_) δ 172.8, 172.7, 160.3, 153.9, 153.9, 149.4, 149.4, 148.1, 148.1, 139.6, 139.3, 137.6, 137.6, 137.5, 135.0, 134.9, 130.0, 128.7, 128.5, 128.5, 127.6, 127.5, 126.4, 126.3, 126.2, 122.7, 122.6, 61.0, 60.9, 48.7, 47.4, 44.9, 44.8, 43.8, 42.5, 41.7, 41.1, 34.1, 27.5, 27.4, 24.9, 24.0, 19.9, 19.9, 19.5, 14.2, 14.2. HRMS (ESI) calcd for C_30_H_36_N_4_O_5_Na [M+Na]^+^: 555.2583, found 555.2592. HPLC purity 100.0% (t_R_ = 12.57 min). HPLC purity 98.54% (t_R_ = 12.83 min).

**Ethyl 3-(2-(3,4-dimethylphenyl)-4-(2-methylbenzyl)-3,5-dioxo-2,3,4,5-tetrahydro-1,2,4-triazine-6-carbonyl)cyclohexane-1-carboxylate (C9).** Compound **C9** was prepared as a white solid (79 mg) in 35% yield from **2h** (155 mg, 0.45 mmol) and ethyl piperidine-3-carboxylate (52 mg, 0.33 mmol). ^1^H NMR (400 MHz, CDCl_3_) δ 7.26 – 7.23 (m, 2H), 7.21 – 7.19 (m, 2H), 7.18 – 7.13 (m, 3H), 5.19 (s, 2H), 4.72 (dd, *J* = 14.2, 3.0 Hz, 0.5H), 4.40 (d, *J* = 13.4 Hz, 0.5H), 4.21 – 4.04 (m, 2H), 3.76 (dd, *J* = 13.8, 3.8 Hz, 0.5H), 3.56 (d, *J* = 13.5 Hz, 0.5H), 3.42 – 3.31 (m, 0.5H), 3.20 – 3.09 (m, 0.5H), 3.10 – 3.02 (m, 0.5H), 3.04 – 2.92 (m, 0.5H), 2.67 – 2.58 (m, 1H), 2.48 (s, 3H), 2.28 (s, 6H), 2.19 – 2.10 (m, 1H), 1.85 – 1.67 (m, 3H), 1.29 – 1.20 (m, 3H). ^13^C NMR (101 MHz, CDCl_3_) δ 172.8, 172.7, 160.3, 154.1, 154.1, 148.2, 139.6, 139.3, 137.7, 137.7, 137.6, 137.5, 136.7, 136.7, 133.1, 133.1, 130.7, 130.7, 130.1, 130.1, 128.0, 128.0, 127.9, 127.9, 126.3, 126.2, 122.7, 122.6, 61.0, 60.9, 48.8, 47.5, 43.9, 42.5, 42.1, 41.7, 41.2, 27.4, 27.4, 24.9, 24.0, 20.0, 19.9, 19.6, 19.6, 14.3. HRMS (EI) calcd for C_28_H_32_N_4_O_5_ [M]^+^: 504.2373, found 504.2380. HPLC purity 97.40% (t_R_ = 12.00 min).

**Ethyl 3-(2-(3,4-dimethylphenyl)-4-(4-methylbenzyl)-3,5-dioxo-2,3,4,5-tetrahydro-1,2,4-triazine-6-carbonyl)cyclohexane-1-carboxylate (C10).** Compound **C10** was prepared as a white solid (80 mg) in 32% yield from **2i** (170 mg, 0.49 mmol) and ethyl piperidine-3-carboxylate (43 mg, 0.27 mmol). ^1^H NMR (400 MHz, CDCl_3_) δ 7.47 – 7.40 (m, 2H), 7.23 (s, 1H), 7.21 – 7.15 (m, 2H), 7.16 – 7.07 (m, 2H), 5.12 (s, 2H), 4.76 – 4.67 (m, 0.5H), 4.47 – 4.37 (m, 0.5H), 4.21 – 4.04 (m, 2H), 3.75 (dd, *J* = 13.5, 4.0 Hz, 0.5H), 3.58 – 3.49 (m, 0.5H), 3.32 (dd, *J* = 13.7, 10.2 Hz, 0.5H), 3.17 – 2.89 (m, 1.5H), 2.68 – 2.54 (m, 1H), 2.32 (s, 3H), 2.28 (s, 6H), 2.23 – 2.09 (m, 1H), 1.86 – 1.62 (m, 3H), 1.30 – 1.18 (m, 3H). ^13^C NMR (101 MHz, CDCl_3_) δ 172.9, 172.8, 160.4, 154.0, 153.9, 148.2, 148.2, 139.7, 139.4, 138.4, 138.3, 137.7, 137.7, 137.6, 137.6, 137.5, 132.2, 132.2, 130.2, 130.1, 129.4, 129.4, 126.4, 126.3, 122.8, 122.7, 61.0, 61.0, 48.8, 47.5, 44.6, 44.6, 43.9, 42.5, 41.8, 41.2, 27.6, 27.4, 25.0, 24.1, 21.3, 20.0, 20.0, 19.6, 14.3, 14.3. HRMS (EI) calcd for C_28_H_32_N_4_O_5_ [M]^+^: 504.2373, found 504.2371. HPLC purity 99.56% (t_R_ = 12.14 min).

**Ethyl 3-(4-(3,5-dimethylbenzyl)-2-(3,4-dimethylphenyl)-3,5-dioxo-2,3,4,5-tetrahydro-1,2,4-triazine-6-carbonyl)cyclohexane-1-carboxylate (C11).** Compound **C11** was prepared as a white solid (70 mg) in 54% yield from **2j** (98 mg, 0.28 mmol) and ethyl piperidine-3-carboxylate (48 mg, 0.31 mmol). ^1^H NMR (300 MHz, CDCl_3_) δ 7.30 – 7.20 (m, 3H), 7.18 (s, 2H), 6.96 (s, 1H), 5.12 (s, 2H), 4.85 – 4.68 (m, 0.5H), 4.46 (d, *J* = 13.5 Hz, 0.5H), 4.28 – 4.04 (m, 2H), 3.80 (dd, *J* = 14.0, 3.8 Hz, 0.5H), 3.59 (d, *J* = 13.4 Hz, 0.5H), 3.36 (dd, *J* = 13.7, 10.2 Hz, 0.5H), 3.23 – 2.91 (m, 1.5H), 2.71 – 2.60 (m, 1H), 2.31 (s, 12H), 2.23 – 2.13 (m, 1H), 1.96 – 1.65 (m, 3H), 1.35 – 1.20 (m, 3H). ^13^C NMR (151 MHz, CDCl_3_) δ 172.8, 172.7, 160.2, 153.8, 153.8, 148.1, 148.0, 143.6, 139.6, 139.3, 137.7, 137.4, 137.4, 136.8, 134.6, 134.5, 130.1, 130.1, 130.0, 130.0, 129.1, 128.8, 128.7, 128.3, 128.3, 126.3, 126.2, 125.4, 122.7, 122.6, 120.4, 109.4, 105.0, 61.7, 61.0, 48.8, 47.5, 44.3, 44.2, 43.9, 42.5, 41.7, 41.2, 29.8, 27.4, 27.4, 24.9, 24.0, 20.0, 20.0, 19.6, 14.3, 14.3. HRMS (ESI) calcd for C_29_H_34_N_4_O_5_Na [M+Na]^+^: 541.2427, found 541.2430. HPLC purity 97.60% (t_R_ = 12.55 min).

**^1^H and ^13^C NMR data**

**C2**

**C3**

**C4**

**C5**

********

**C6**

**C7**

**C8**

**C9**

**C10**

**C11**
